# Global proteomic analyses define an environmentally contingent Hsp90 interactome and reveal chaperone-dependent regulation of stress granule proteins and the R2TP complex in a fungal pathogen

**DOI:** 10.1371/journal.pbio.3000358

**Published:** 2019-07-08

**Authors:** Teresa R. O’Meara, Matthew J. O’Meara, Elizabeth J. Polvi, M. Reza Pourhaghighi, Sean D. Liston, Zhen-Yuan Lin, Amanda O. Veri, Andrew Emili, Anne-Claude Gingras, Leah E. Cowen

**Affiliations:** 1 Department of Molecular Genetics, University of Toronto, Toronto, Canada; 2 Department of Pharmaceutical Chemistry, University of California, San Francisco, San Francisco, California, United States of America; 3 Donnelly Centre for Cellular and Biomolecular Research, University of Toronto, Toronto, Canada; 4 Lunenfeld-Tanenbaum Research Institute, Sinai Health System, Toronto, Canada; 5 Department of Biology, Boston University School of Medicine, Boston, Massachusetts, United States of America; 6 Department of Biochemistry, Boston University School of Medicine, Boston, Massachusetts, United States of America; Academia Sinica, TAIWAN

## Abstract

Hsp90 is a conserved molecular chaperone that assists in the folding and function of diverse cellular regulators, with a profound impact on biology, disease, and evolution. As a central hub of protein interaction networks, Hsp90 engages with hundreds of protein–protein interactions within eukaryotic cells. These interactions include client proteins, which physically interact with Hsp90 and depend on the chaperone for stability or function, as well as co-chaperones and partner proteins that modulate chaperone function. Currently, there are no methods to accurately predict Hsp90 interactors and there has been considerable network rewiring over evolutionary time, necessitating experimental approaches to define the Hsp90 network in the species of interest. This is a pressing challenge for fungal pathogens, for which Hsp90 is a key regulator of stress tolerance, drug resistance, and virulence traits. To address this challenge, we applied a novel biochemical fractionation and quantitative proteomic approach to examine alterations to the proteome upon perturbation of Hsp90 in a leading human fungal pathogen, *Candida albicans*. In parallel, we performed affinity purification coupled to mass spectrometry to define physical interacting partners for Hsp90 and the Hsp90 co-chaperones and identified 164 Hsp90-interacting proteins, including 111 that are specific to the pathogen. We performed the first analysis of the Hsp90 interactome upon antifungal drug stress and demonstrated that Hsp90 stabilizes processing body (P-body) and stress granule proteins that contribute to drug tolerance. We also describe novel roles for Hsp90 in regulating posttranslational modification of the Rvb1-Rvb2-Tah1-Pih1 (R2TP) complex and the formation of protein aggregates in response to thermal stress. This study provides a global view of the Hsp90 interactome in a fungal pathogen, demonstrates the dynamic role of Hsp90 in response to environmental perturbations, and highlights a novel connection between Hsp90 and the regulation of mRNA-associated protein granules.

## Abstract 2

A global view of the Hsp90 chaperone interactome in the fungal pathogen Candida albicans reveals the dynamic role of Hsp90 in response to environmental perturbations, and highlights a novel connection between Hsp90 and the regulation of mRNA-associated protein granules.

## Introduction

All organisms must maintain protein homeostasis, despite remarkable intracellular macromolecular crowding and exposure to environmental stressors that perturb protein folding. Elevated temperature is a ubiquitous stress that causes protein damage and misfolding, thereby disrupting protein function and exposing hydrophobic residues that can trigger aggregation [1]. The resulting stress-dependent aggregates are comprised of protein/mRNA-associated granules known as heat shock granules, processing bodies (P-bodies), or stress granules, depending on the proteins present [2–4]. These aggregates are thought to function as a mechanism for attenuating translation [5,6] and protecting cells during periods of stress [2], suggesting that the formation and disaggregation of these granules must be a regulated process. Thus far, the Hsp104 disaggregase and the Hsp70 chaperone protein are known to aid in a disassembly of aggregates upon a return to non-stressful conditions in *Saccharomyces cerevisiae* [5]. Other chaperone proteins are likely to assist in assembly or disaggregation of granules due to their ability to transiently bind other proteins to aid in their folding, function, trafficking, and degradation [7], but the roles of these other chaperones have not yet been defined.

A core hub of protein homeostasis networks in eukaryotes is the heat shock protein Hsp90, a conserved and essential molecular chaperone that assists in protein folding and activation, protein complex assembly, and protein ligand binding [8–10]. Many of the client proteins that interact with Hsp90 and depend on the chaperone for proper form and function are kinases, transcription factors, or other signalling proteins, thus placing Hsp90 at the center of many signal transduction networks [8,11]. Hsp90 is also involved in loading small RNAs onto Argonaute proteins, thus regulating the RNA-induced silencing complex [12,13] and implicating Hsp90 in RNA-protein complex assembly. Hsp90 function is regulated both by posttranslational modifications and by dynamic interactions with co-chaperones that influence conformational changes associated with the chaperone cycle [8]. As a consequence of its key roles in regulating protein homeostasis and cellular signaling, Hsp90 has a profound impact on diverse facets of biology and disease, and modulates the translation of genotype to phenotype and the capacity to evolve new traits [11,14].

Fungi provide tractable model systems to both explore the impact of Hsp90 on genotype to phenotype relationships and define chaperone networks [15–18]. Targeting the fungal chaperone network is also a promising approach to mitigate a serious threat to human health [19,20]. Fungal pathogens kill as many people as malaria and tuberculosis worldwide [21], and are a growing public health threat with the pervasive evolution of resistance to our current limited arsenal of antifungal drugs [22]. In a leading human fungal pathogen, *Candida albicans*, impairment of Hsp90 function abrogates drug resistance, reduces tolerance to a myriad of stresses, induces a morphological transition from yeast to filamentous growth, and attenuates virulence [19,23,24]. Although targeting Hsp90 may provide a powerful strategy to cripple fungal pathogens, and diverse Hsp90 inhibitor chemotypes have been explored in anticancer programs [25], the antifungal utility of current Hsp90 inhibitors is compromised by toxicity due to concurrent inhibition of the host chaperone protein [26]. Thus, it is critical to identify fungal-selective Hsp90 inhibitors or components of the Hsp90 chaperone network that enable stress responses, drug resistance, and virulence, and that are sufficiently divergent to enable selective targeting in the pathogen. Only a few Hsp90 client proteins important for these traits have been identified in *C*. *albicans*, including the Hog1 mitogen-activated protein kinase (MAPK); components of the Protein Kinase C (PKC) cell wall integrity cascade (Pkc1, Bck1, Mkk1, and Mkc1); the catalytic subunit of the calcineurin phosphatase, Cna1; the Cka2 casein kinase; the Cdc28 cyclin-dependent kinase; the Hsf1 transcription factor; the Cdr1 transporter; and the Cyr1 adenylyl cyclase protein [27–32]. Despite previous comprehensive analyses of Hsp90 networks in the model yeast *S*. *cerevisiae*, the established divergence in Hsp90 chemical genetic interactions, gene essentiality, and gene function between the species [28,33] highlights the importance of directly characterizing the Hsp90 chaperone network in the pathogen.

Here, we define the global proteomic changes in response to perturbation of protein homeostasis and map the Hsp90 interaction network in *C*. *albicans*. We used a biochemical fractionation and quantitative proteomic approach to monitor the impact of Hsp90 perturbation on the proteome, and leveraged affinity purification and mass spectrometry to identify Hsp90 physical interaction partners. We also examined how the interaction network changes upon environmental perturbations, such as exposure to antifungal drug stress. In our downstream analyses, we evaluated the functional dependence of novel interactors on Hsp90, establishing Pbs2 as a new Hsp90 client that is influenced by environmental stress. Our analyses also revealed a role for Hsp90 in the regulation of stress-dependent posttranslational modification of Rvb1, a member of the Rvb1-Rvb2-Tah1-Pih1 (R2TP) complex. We further demonstrate that Hsp90 regulates the stability of stress granule and P-body proteins, including Dhh1, Kre30, and Cam1, and establish a connection between Hsp90 dependence and the formation of aggregates under thermal stress. Additionally, these stress granule and P-body proteins contribute to antifungal drug tolerance. Together, this greatly expands our knowledge of how Hsp90 regulates cellular stress responses and defines a protein homeostasis network in a human fungal pathogen.

## Results

### Defining the Hsp90 interactome in *C*. *albicans*

To characterize the impact of Hsp90 on the proteome and define the Hsp90 interactome, we used a combination of targeted affinity purification-mass spectrometry (AP-MS) and whole-cell quantitative proteomic approaches to identify proteins that interact with Hsp90 and depend on Hsp90 for stability. We first examined the physical interactors of Hsp90 by performing AP-MS analysis of *C*. *albicans* Hsp90 and Hsp90 co-chaperones under standard laboratory growth conditions. In *S*. *cerevisiae*, identification of interactions between Hsp90 and client proteins was aided by the use of an E33A mutant of Hsp90 that effectively traps client proteins by decreasing Hsp90 ATPase activity [34,35]. We created the analogous heterozygous mutant (E36A) of Hsp90 in *C*. *albicans* and then added a C-terminal tandem affinity purification (TAP) or green fluorescent protein (GFP) tag. To confirm that the tag and E36A substitution did not interrupt normal dimerization of Hsp90, we performed immunoprecipitation of the GFP-tagged Hsp90 proteins and demonstrated that the tagged proteins co-purified with untagged Hsp90 in western immunoblots (S1A Fig). Similarly, an N-terminally tagged Hsp90 protein also co-purified untagged Hsp90 (S1B Fig). Additionally, we generated C-terminally TAP-tagged versions of nine Hsp90 co-chaperones (Aha1, Cdc37, Cpr6, Cpr7, Cns1, Hch1, Sba1, Sti1, and Sgt1) to more broadly map the Hsp90 interactome. We affinity purified each co-chaperone using immunoglobulin G (IgG) beads followed by tobacco etch virus (TEV)-protease cleavage and a second round of purification using calmodulin beads. To distinguish interactors from background, we used Significance Analysis of INTeractome (SAINTexpress) to compare the interactors identified with those identified with an unrelated TAP-tagged protein, Cas5, using a false discovery rate of 2% [36].

**Fig 1:**
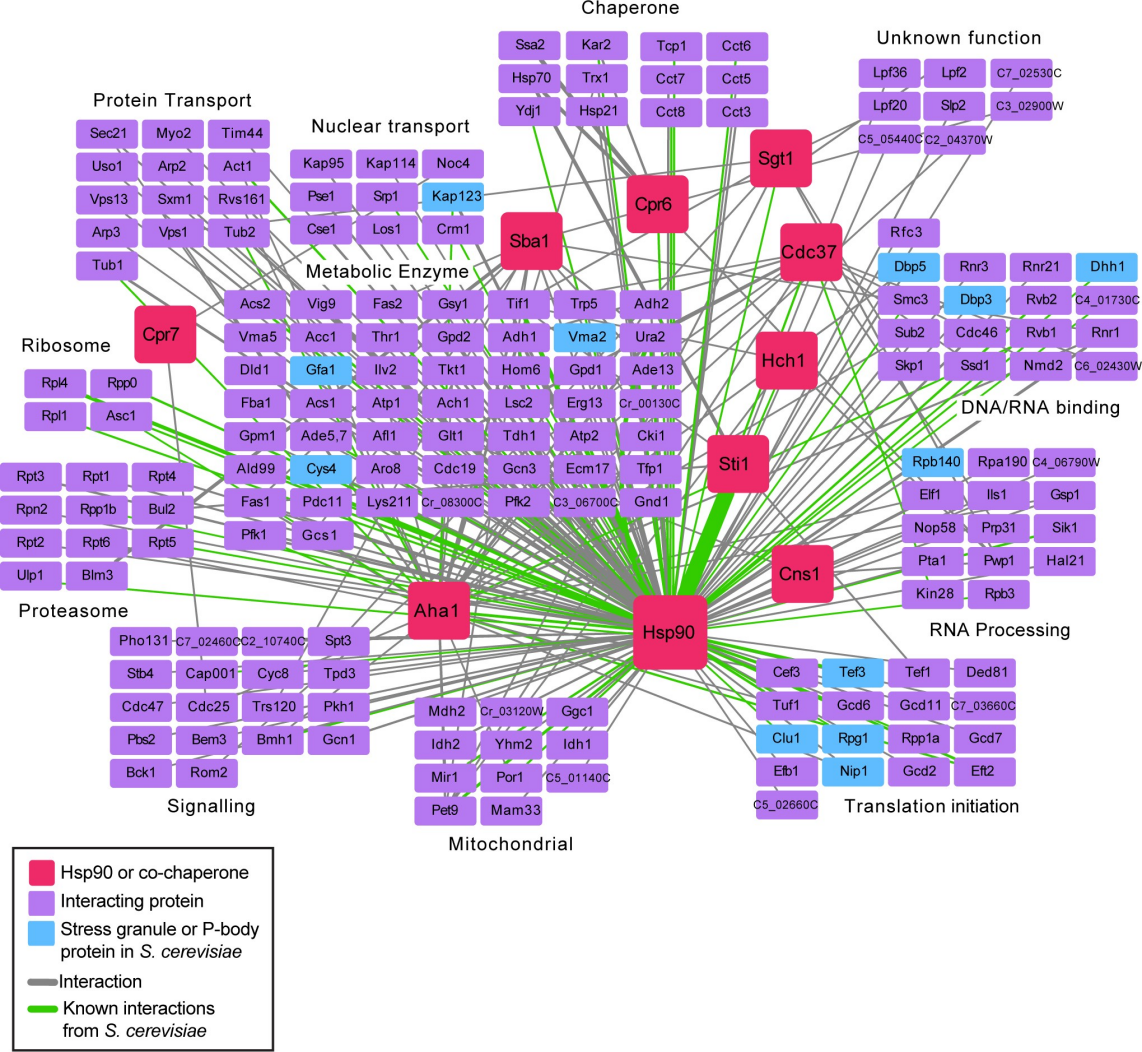
AP-MS approaches identify novel interactors for *C*. *albicans* Hsp90. AP-MS of affinity-tagged Hsp90^E36A^ and nine Hsp90 co-chaperones identified physically interacting proteins. Cells were grown in YPD at 30°C, and statistically significant interactions were defined through SAINTexpress analysis compared with an unrelated tagged protein. Pink squares represent the 10 bait proteins. Purple squares represent prey proteins that were statistically significant interactors by SAINTexpress, and are grouped based on GO term annotation. Blue squares represent known stress granule or P-body proteins in *S*. *cerevisiae*. The width of the edges corresponds to peptide counts (peptide counts provided in S1 Table), and green edges represent physical interactions that have also been previously identified in *S*. *cerevisiae*, as annotated in BioGRID. AP-MS, affinity purification-mass spectrometry; GO, gene ontology; P-body, processing body; SAINTexpress, Significance Analysis of INTeractome; YPD, yeast extract peptone dextrose.

Through this analysis, we identified 256 significant associations for Hsp90 and the Hsp90 co-chaperones, covering 188 unique proteins (Fig 1, S1 Table). Of these, only 65 have biological general repository for interaction dataset (BioGRID) annotations for physical interactions with Hsp90 or the nine Hsp90 co-chaperones in *S*. *cerevisiae* (S1 Table). We then sorted the Hsp90 physical interaction dataset into functional categories based on gene ontology (GO) term mapping and hand annotation (Fig 1). We categorized Hsp90 interactors into clusters comprised of proteins involved in RNA binding and metabolism, cellular transport, metabolism, protein fate, and signalling, highlighting the diverse cellular processes impacted by Hsp90.

Notably, we were not able to observe interactions between Hsp90 and all of the Hsp90 co-chaperones through this approach. One hypothesis is that although the C-terminal tag on Hsp90 does not prevent Hsp90 dimerization or function, it could interfere with proteins binding to the Hsp90 tetratricopeptide repeat (TPR) domain [15]. For example, the C-terminally tagged Hsp90 protein did not identify the Sti1 co-chaperone as an interactor (S1 Table), presumably because this protein binds to the TPR domain at the C terminus of Hsp90 [37]. Using TAP-tagged Sti1 as the bait, however, we identified untagged Hsp90 as a strong interactor (Fig 1, S1 Table), highlighting the utility of the co-chaperone interactors in defining the Hsp90 network in *C*. *albicans*.

As an orthogonal approach to examine the Hsp90 interactome in *C*. *albicans*, we examined the proteome in response to perturbations to Hsp90. To allow for a quantitative and multiplexed analysis, we used stable isotope labeling with amino acids in cell culture (SILAC) experiments to differentially label each sample. This experiment required auxotrophies to adequately label the proteins with stable isotopes; therefore, we generated a lysine auxotroph by deleting both alleles of *LYS2* from the diploid wild-type parental strain. We then used unlabeled L-lysine (light), ^13^C_6_,^15^N_2_-L-lysine (heavy), or 4,4,5,5-D_4_-L-Lysine (medium) to differentially label samples. For genetic control of *HSP90*, we first deleted one allele and placed the remaining allele under control of a tetracycline-repressible *tetO* promoter in the *lys2*Δ*/lys2*Δ strain background. This strain was then treated with the tetracycline analog doxycycline (DOX) for transcriptional repression of *HSP90* (S2A Fig). As a complementary approach to compromise Hsp90 function, we used a potent and specific natural product inhibitor, geldanamycin (GdA), at 15 μM. These treatments did not impair viability but induced a morphological transition from yeast to filaments, consistent with inhibition of Hsp90 function (S2B Fig) [19,24]. We then performed deep fractionation of the soluble protein extracts under non-denaturing conditions using ion exchange–high performance liquid chromatography (IEX-HPLC), generating 120 fractions per sample. These methods were developed to maintain protein complexes and protein–protein interactions [38]. Each sample was then examined by nano-flow liquid chromatography–tandem mass spectrometry (LC-MS/MS) to identify the proteins present in each collected fraction.

**Fig 2:**
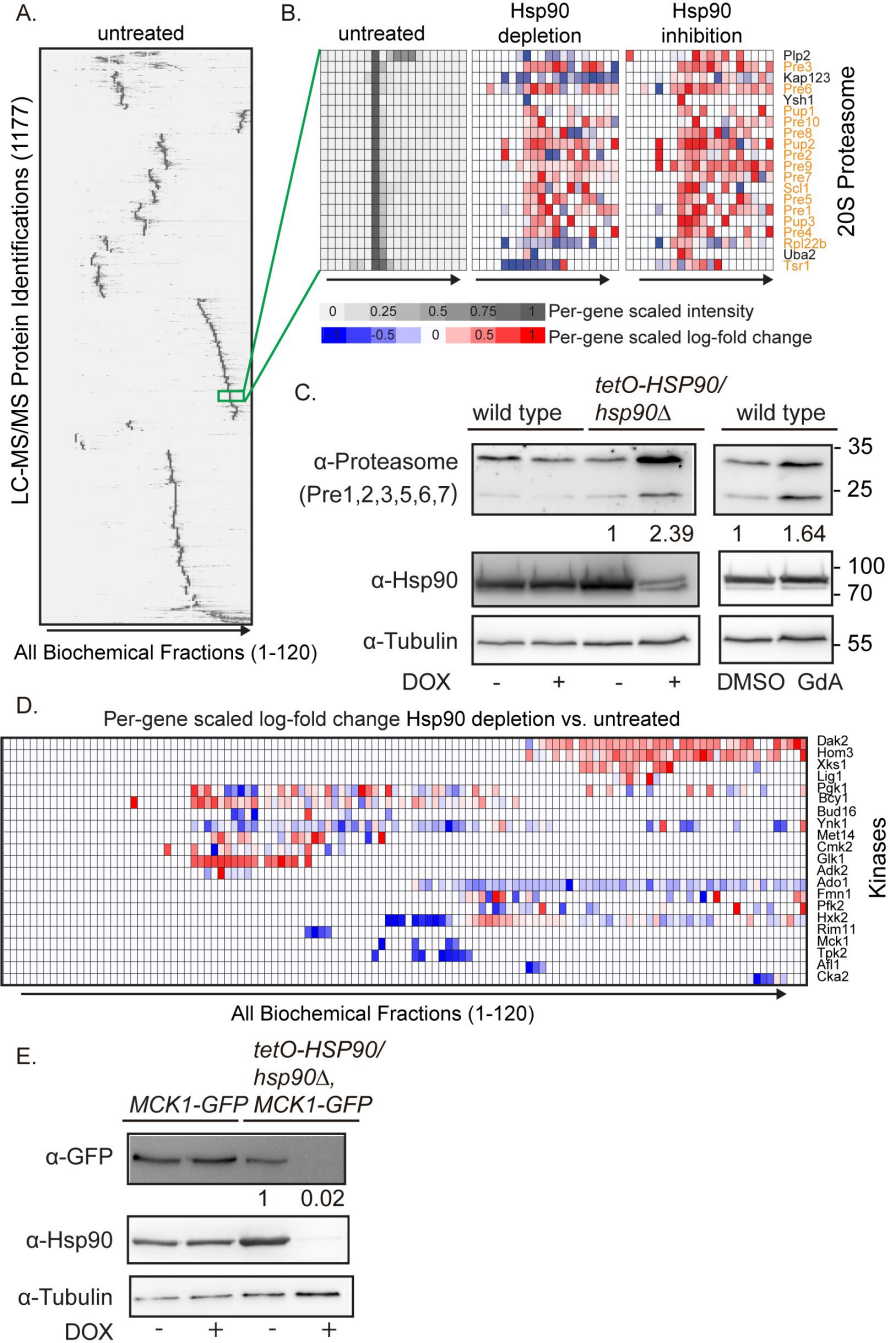
Global proteomic changes upon perturbation of Hsp90. (A) Global proteome analysis in *C*. *albicans*. Non-denaturing IEX fractionation and MS experiments were performed on *lys2Δ/lys2Δ tetO-HSP90/hsp90Δ* cells that were untreated, treated with DOX to repress *HSP90* expression, or treated with 15 μM GdA to inhibit Hsp90 function. For DOX treatment, cells were treated with 0.05 μg/mL DOX overnight and then subcultured into medium with 5 μg/mL DOX for 4 hours before harvesting. Displayed is the co-fractionation (IEX-HPLC) profile of the *C*. *albicans* untreated extracts. Shading for the untreated condition indicates spectral counts recorded in LC-MS/MS, with intensity normalized on a per-gene basis. Clustering order maximizes the correlation of adjacent intensity profiles from the untreated sample. Raw data for this figure can be found in S2 Table. (B) Co-fractionation (IEX-HPLC) reveals a 20S proteasome cluster. This co-fractionation cluster is enriched for the 20S proteasome, with proteins in this category highlighted in orange. Shading for the untreated condition indicates spectral counts recorded in LC-MS/MS, with intensity normalized on a per-gene basis. For the treatment conditions, shading indicates per-gene scaled log-fold change compared with the untreated sample, with blue indicating a decrease and red indicating an increase in peptide abundance. Raw data for this figure can be found in S2 Table and S3 Table. (C) Proteasome protein levels increase upon Hsp90 perturbation. To repress Hsp90, cells were grown overnight in ±0.5 μg/mL DOX to repress *HSP90* expression in the *tetO-HSP90/hsp90Δ* strain and then subcultured into medium ± 5 μg/mL DOX for 4 hours. For pharmacological inhibition of Hsp90, wild-type cells were grown overnight in YPD and then subcultured into medium with 15 μM GdA or DMSO vehicle control for 4 hours. Proteins were extracted and immunoblotted with an antibody specific to a conserved peptide in the 20S proteasome proteins Pre1 (22.0 kDa), Pre2 (31.2 kDa), Pre3 (23.3 kDa), Pre5 (31.4 kDa), Pre6 (27.4 kDa), and Pre7 (27.4 kDa). Protein levels were normalized to the tubulin loading control and quantified compared with their respective vehicle controls. (D) Alteration in kinase levels upon Hsp90 depletion. Protein abundance for predicted kinase proteins that showed >1.5-fold log change in abundance across two or more fractions in cells with DOX-mediated transcriptional repression of *HSP90* compared with untreated cells in the *tetO-HSP90/hsp90Δ* strain. Shading indicates per-gene scaled log-fold change compared with the untreated sample, with blue indicating a decrease and red indicating an increase. Raw data for this figure can be found in S3 Table. (E) Mck1 protein levels decrease upon transcriptional repression of *HSP90*. Cells were grown overnight in ±0.5 μg/mL DOX to repress *HSP90* in the *tetO-HSP90/hsp90Δ* strain and then subcultured into medium ± 5 μg/mL DOX for 4 hours before protein extraction and western blotting. Protein levels were normalized to the tubulin loading control and quantified compared with the vehicle controls. DOX, doxycycline; GdA, geldanamycin; IEX-HPLC, ion exchange–high performance liquid chromatography; LC-MS/MS, liquid chromatography–tandem mass spectrometry; YPD, yeast extract peptone dextrose.

Using this approach, we identified 1,177 distinct proteins, covering approximately 20% of the *C*. *albicans* proteome in the untreated sample (Fig 2A, S2 Table). This co-fractionation and LC-MS/MS approach had been used to identify stably interacting protein complexes based on co-elution in humans [38], but has not been previously attempted for a fungal pathogen. From co-elution data, it is possible to identify new protein complexes without prior knowledge of complex formation, making it a useful tool to explore understudied organisms. For each of the conditions, the Spearman rank correlation of intensities, averaging ties, was computed for each pair of genes using the EGAD R package [39]. Taking the correlations for each condition as a network, the proteins were then clustered using affinity propagation using the apcluster R package [40]. Using this method, we identified 37 putative co-eluting clusters, which potentially represent protein complexes (S2 Table). As an example, we identified a cluster comprised of each component of the 20S proteasome (Fig 2B). Together, these data highlight our ability to identify putative stable protein complexes based on co-fractionation.

Next, we compared the intensity, elution profile, and clustering of the identified proteins upon alterations in proteostasis induced by *HSP90* genetic depletion or inhibition, as compared with the untreated control sample. We identified 400 proteins for which abundance was altered by at least 1.5-fold in at least two fractions upon both Hsp90 inhibition and depletion (S3 Table). As an example, we observed that both depletion and inhibition of Hsp90 resulted in increased levels of 20S proteasome proteins (Fig 2B). To examine this set in more detail, we examined proteasome protein levels in response to Hsp90 depletion in the *tetO-HSP90/hsp90*Δ strain or in response to treatment with GdA by western blotting. In the DOX-treated *tetO-HSP90/hsp90*Δ cells or the GdA-treated wild-type cells, we observed an increase in proteasome protein levels compared with vehicle controls (Fig 2C), validating these SILAC protein quantifications.

In addition to changes in entire protein complexes, we can also examine specific proteins to determine their abundance upon depletion of Hsp90. Because of the established role of Hsp90 in stabilizing kinases, we examined the profiles of kinases identified in the proteomic analyses and observed 21 kinases with a >1.5-fold change in abundance upon Hsp90 depletion (Fig 2D, S3 Table). To examine an example kinase in more detail, we created a GFP-tagged version of the Mck1 dual specificity kinase protein in the wild-type and *tetO-HSP90/hsp90*Δ strains. Upon Hsp90 depletion with DOX, we observed that the Mck1-GFP protein decreased in abundance (Fig 2E), demonstrating that Mck1 depends on Hsp90 for stability. From our combination of AP-MS approaches and proteome-level depletion quantifications, we identified proteins that both interact with Hsp90 and depend on Hsp90 for stability. In sum, we mapped the Hsp90 physical interaction network and defined proteomic changes upon Hsp90 inhibition.

### The Hsp90 interactome is modulated by stress

A key feature of the Hsp90 genetic interaction network is that it is environmentally contingent, with many genetic interactions only observed in the presence of an additional stressor [17,28,33]. However, for protein–protein interactions to change, there must be alterations in protein abundance, localization, or biochemical properties that could change the ability of two proteins to interact. In the model yeast *S*. *cerevisiae*, changes in Hsp90 interactors in response to stress have only been examined after treating cells with methyl methanesulfonate (MMS) as a DNA-damaging stress, and in this study, relatively few proteins changed in their interaction with Hsp90 [41]. Because of the importance of Hsp90 for antifungal drug tolerance, we examined whether the Hsp90 interactome was altered upon exposure to two different antifungal drugs with distinct modes of action. For these experiments, we incubated cells with either the azole antifungal drug fluconazole, which targets biosynthesis of the membrane sterol ergosterol, or with the echinocandin antifungal drug caspofungin, which inhibits biosynthesis of cell wall ß-1,3-glucan. Both antifungals were used at concentrations that inhibit growth by approximately 20%. We then performed affinity purification of Hsp90^E36A^-GFP using “GFP-trap” resin, followed by mass spectrometry. This single-step purification allows for more quantitative measurements of changes in the Hsp90 interactome. To distinguish interactors from background, we compared interactors identified with Hsp90^E36A^-GFP with those identified with a control Eno1-GFP using SAINTexpress and a Bayesian false discovery rate (BFDR) cutoff of 2% to define significant interactors (S4 Table).

**Fig 3:**
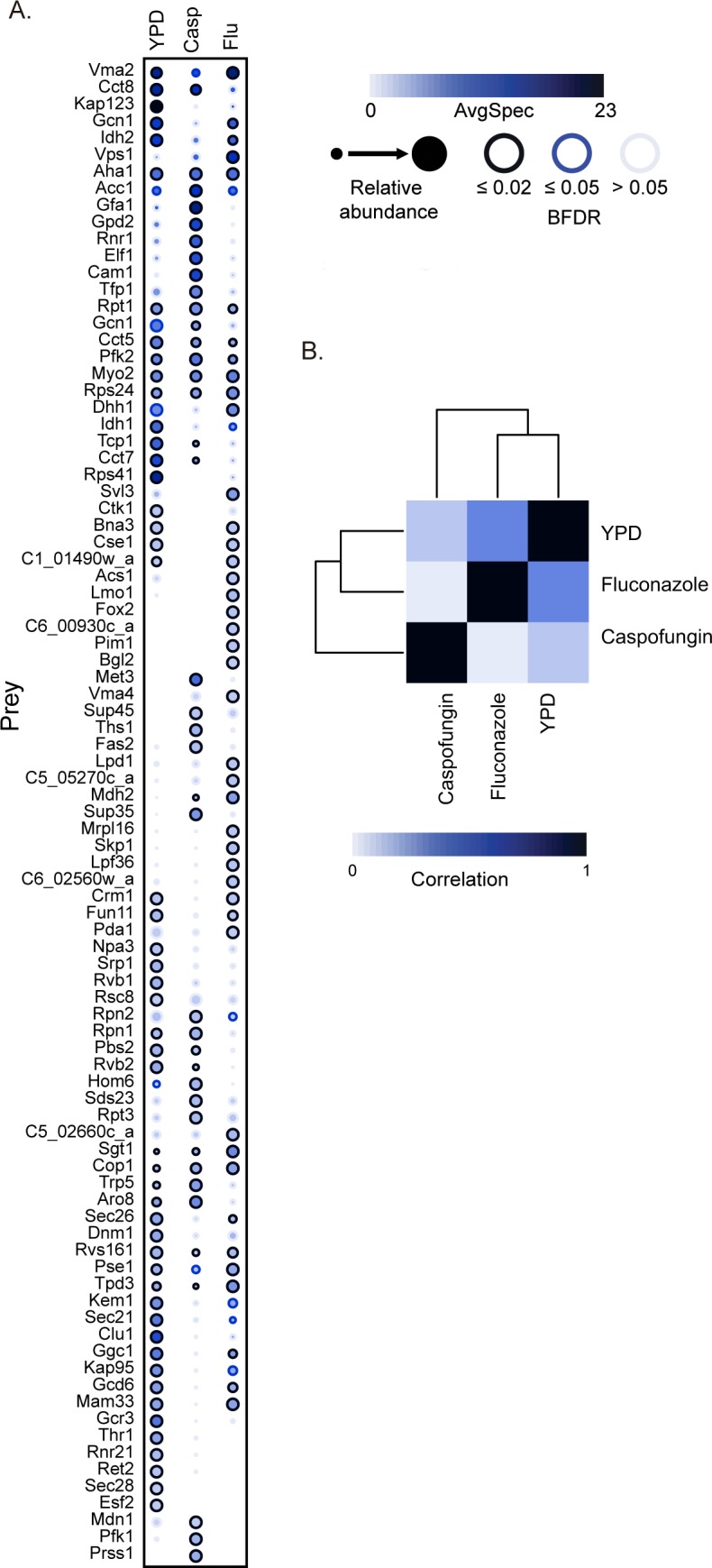
The Hsp90 physical interaction network is modulated by antifungal drug stress. (A) AP-MS was performed on *HSP90*^*E36A*^*-GFP/HSP90* cells grown at 30°C in the presence or absence of 8 μg/mL fluconazole (Flu) or 100 nM caspofungin (Casp). Statistically significant Hsp90 interaction partners are shown as a dot plot in which node color represents the absolute number of spectral counts, node size represents relative abundance between samples, and node edges represent the SAINTexpress BFDR rate at which a given prey protein was observed. Raw data for this figure can be found in S4 Table. (B) Bait versus bait comparison shows the highest correlation is between interactors identified in the YPD and fluconazole conditions. Color scale represents the degree of correlation. AP-MS, affinity purification-mass spectrometry; AvgSpec, Average Spectral Count; BFDR, Bayesian false discovery rate; GFP, green fluorescent protein; SAINTexpress, Significance Analysis of INTeractome.

We then examined the correlation in prey abundance of the significant physical interactors between the drug-treated and untreated samples (Fig 3A, S3 Fig). We identified 16 proteins that had at least a 2-fold change in peptide abundance upon drug treatment and many that were only significant interactors with Hsp90 under drug stress conditions. Overall, we observed a stronger correlation in peptide abundance between the untreated and fluconazole-treated samples (*r*^2^ = 0.4185) than between the untreated and caspofungin-treated samples (*r*^2^ = 0.1208) (Fig 3B, S3 Fig). The difference between the Pearson correlation coefficients is 0.29, which is statistically significant under the Fisher z-test (*P* < 0.0075), and Zou's 95% confidence interval (0.078–0.52) excludes 0 [42,43]. One signature was increased pulldown of translational regulators such as Sup35, Sup45, and Cam1 in response to caspofungin stress, but not in response to fluconazole. Together, these data demonstrate that there are shifts in the physical interactome of Hsp90 in response to antifungal drug treatment, suggesting that Hsp90 plays different roles in regulating proteostasis in response to different environmental perturbations.

### Identification of a novel Hsp90 client protein in *C*. *albicans*

**Fig 4:**
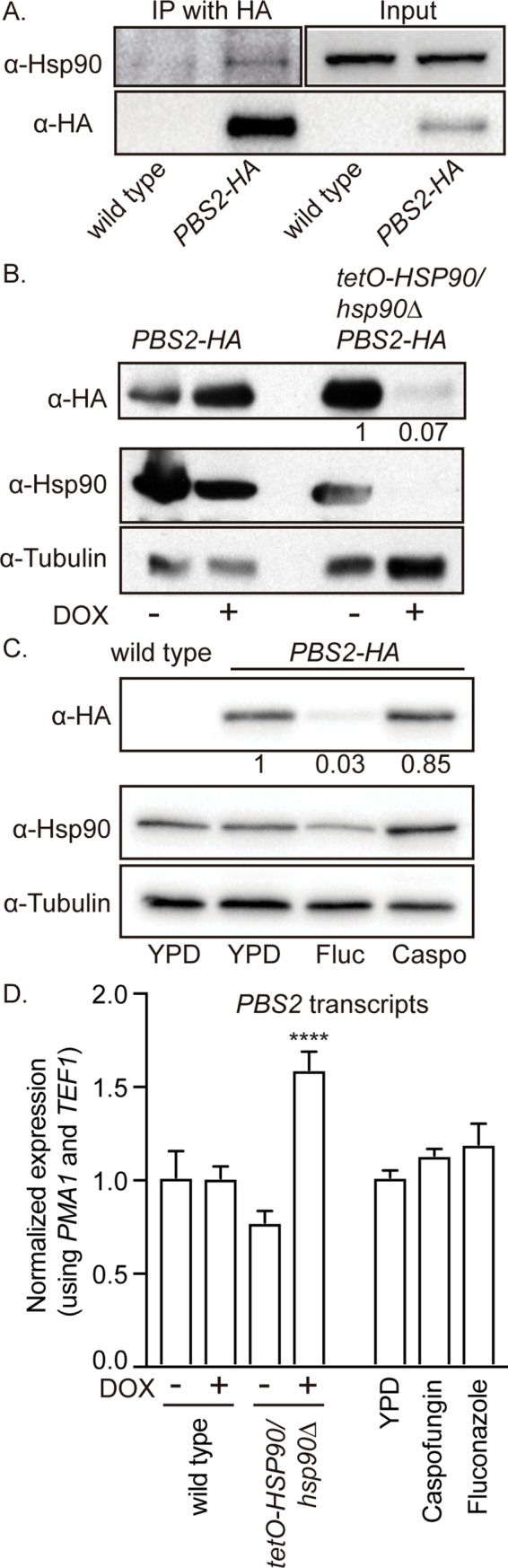
Pbs2 is a client of Hsp90 in *C*. *albicans*. (A) Pbs2 and Hsp90 physically interact. Immunoprecipitation of Pbs2-HA with anti-HA agarose co-purified Hsp90, while Hsp90 did not co-purify with anti-HA agarose in control cells lacking tagged Pbs2. There was no difference in Hsp90 levels between input samples. (B) Pbs2-HA is destabilized upon transcriptional repression of *HSP90*. Cells were grown overnight in ±0.5 μg/mL DOX to repress *HSP90* in the *tetO-HSP90/hsp90Δ* strain and then subcultured into medium ± 5 μg/mL DOX for 4 hours before protein extraction and western blotting. Protein levels were normalized to the tubulin loading control and quantified compared with the no DOX control. (C) Pbs2-HA protein levels decrease upon fluconazole but not caspofungin stress. Cells were incubated with 8 μg/mL fluconazole (Fluc) or 100 nM caspofungin (Caspo) before protein extraction and western blotting. Protein levels were normalized to the tubulin loading control and quantified compared with the no drug control. (D) *PBS2* transcript levels are not altered by transcriptional repression of *HSP90* or treatment with antifungal drugs. Cells were grown overnight in ±0.5 μg/mL DOX to repress *HSP90* in the *tetO-HSP90/hsp90Δ* strain and then subcultured into medium ± 5 μg/mL DOX for 4 hours before RNA extraction and qRT-PCR. Cells were incubated with 8 μg/mL fluconazole or 100 nM caspofungin before RNA extraction and qRT-PCR. *PBS2* transcript levels were normalized to *PMA1* and *TEF1*. Significance was determined by one-way ANOVA. **** indicates P-value < 0.001. Raw data for this figure can be found in S1 Data. Caspo, caspofungin; DOX, doxycycline; Fluc, fluconazole; HA, hemagglutinin; IP, immunoprecipitation; qRT-PCR, quantitative reverse transcription PCR; YPD, yeast extract peptone dextrose.

We leveraged our analysis of the Hsp90 interactome to define Hsp90 client proteins in *C*. *albicans*. One interesting candidate was Pbs2, which is the kinase directly upstream of Hog1 in the Hog1 MAPK cascade [44]. Hog1 is an established client of Hsp90 in both *S*. *cerevisiae* and *C*. *albicans*, and we have previously observed that Hsp90 can chaperone multiple steps of a single kinase cascade [27]. We found that Pbs2 decreased in abundance in Hsp90 pulldowns with fluconazole treatment (Fig 3A). We validated that endogenous untagged Hsp90 and C-terminally hemagglutinin (HA)-tagged Pbs2 physically interact by co-immunoprecipitation using anti-HA agarose (Fig 4A). To determine if Pbs2 stability depends on Hsp90, we HA-tagged Pbs2 in the *tetO-HSP90/hsp90*Δ strain and monitored Pbs2 levels by western blotting upon transcriptional repression of *HSP90* with DOX. Depletion of Hsp90 was accompanied by a striking reduction in Pbs2-HA levels (Fig 4B). This occurred despite an increase in *PBS2* transcripts, demonstrating that the decrease in Pbs2 occurs at the protein level (Fig 4D). Treatment with fluconazole was also sufficient to reduce Pbs2-HA levels, while treatment with caspofungin had no effect (Fig 4C). The reduction in Pbs2-HA levels upon fluconazole treatment was not due to a change in transcript levels, as quantitative reverse transcription PCR (qRT-PCR) experiments revealed no significant changes in *PBS2* levels upon drug treatment (Fig 4D). We had previously observed that membrane stress conditions, such as alterations in ergosterol biosynthesis, can overwhelm the functional capacity of Hsp90 [33]; this is consistent with the finding that the altered interaction between Hsp90 and Pbs2 was specific to membrane stress conditions. Together, this defines Pbs2 as an Hsp90 client in *C*. *albicans* and highlights the environmental contingency of Hsp90 interactions.

### Sumoylation of Rvb1 is repressed by Hsp90

**Fig 5:**
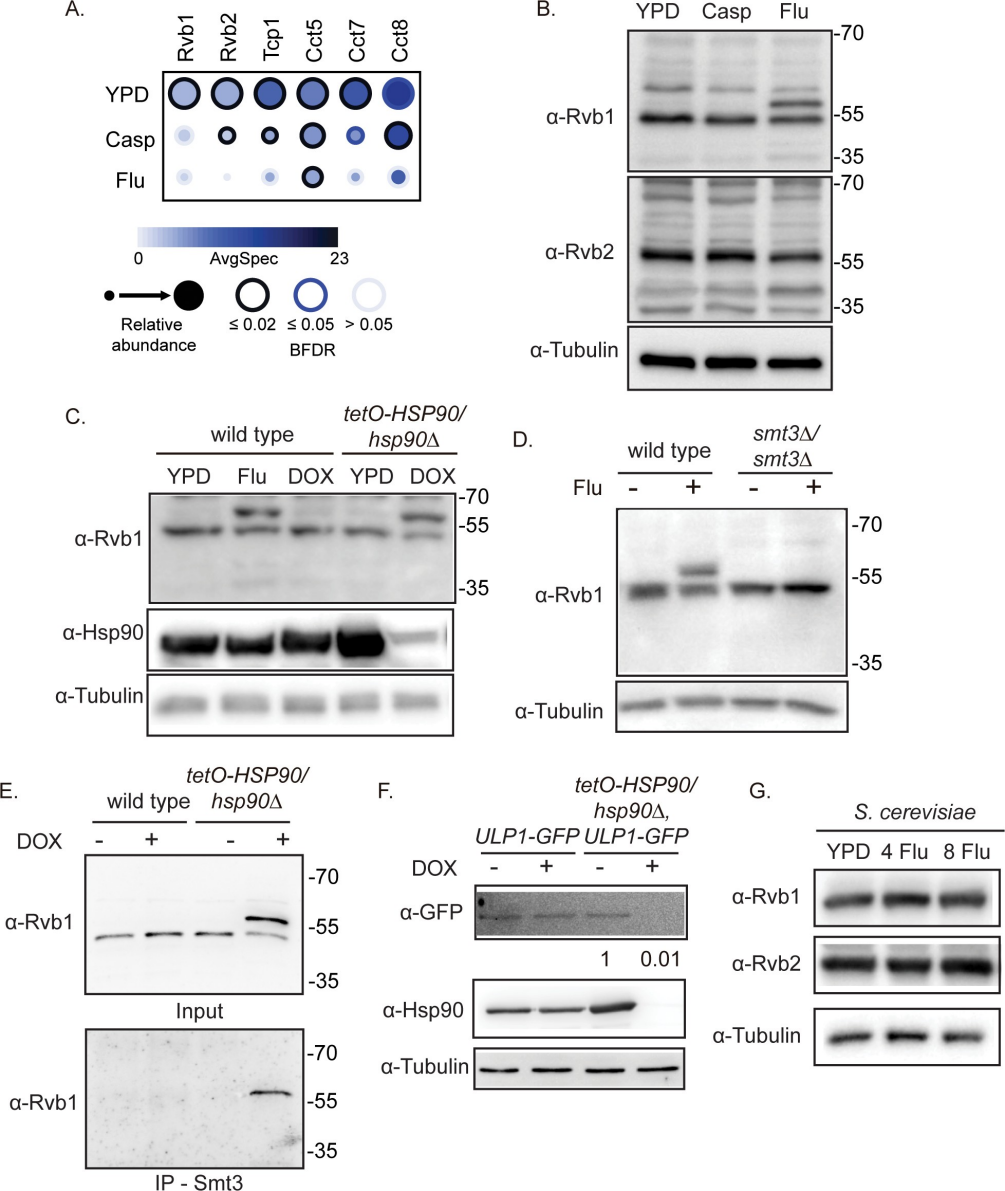
Hsp90 represses the sumoylation of Rvb1. (A) Chaperone proteins vary in their interaction with Hsp90 upon drug treatment. AP-MS was performed on *HSP90*^*E36A*^*-GFP/HSP90* cells grown at 30°C in the presence or absence of 8 μg/mL fluconazole (Flu) or 100 nM caspofungin (Casp). Hsp90 interaction partners are shown as a dot plot in which the node color represents the absolute spectral count, node size represents relative abundance between the conditions, and node edge color represents the SAINTexpress BFDR rate. Raw data for this figure can be found in S4 Table. (B) Rvb1 migrates as two bands upon fluconazole treatment. Cells were incubated with 8 μg/mL fluconazole (Flu) or 100 nM caspofungin (Casp) before protein extraction and western blotting. Rvb2 migration is unaffected by antifungal drug treatment. Molecular weight markers are shown (kDa). (C) Rvb1 migrates as two bands upon Hsp90 depletion. Wild-type and *tetO-HSP90/hsp90Δ* cells were grown overnight in ±0.5 μg/mL DOX to repress *HSP90* in the *tetO-HSP90/hsp90Δ* strain and then subcultured into medium ± 5 μg/mL DOX for 4 hours before protein extraction and western blotting. Wild-type cells were also incubated with 8 μg/mL fluconazole (Flu). Molecular weight markers are shown (kDa). (D) The change in Rvb1 migration depends on the Smt3 SUMO protein. Cells were incubated with or without 8 μg/mL fluconazole (Flu) before protein extraction and western blotting. Molecular weight markers are shown (kDa). (E) Rvb1 is sumoylated. Smt3-specific antibodies immunoprecipitate the high-molecular weight band of Rvb1. Cells were grown overnight in ±0.5 μg/mL DOX to repress *HSP90* in the *tetO-HSP90/hsp90Δ* strain and then subcultured into medium ± 5 μg/mL DOX for 4 hours before protein extraction. Samples were immunoprecipitated with an anti-Smt3 antibody and probed with an anti-Rvb1 antibody. The anti-Rvb1 antibody only identified a band upon Hsp90 depletion. Molecular weight markers are shown (kDa). (F) Ulp1-GFP is destabilized upon transcriptional repression of *HSP90*. Wild-type and *tetO-HSP90/hsp90Δ* cells were grown overnight in ±0.5 μg/mL DOX to repress *HSP90* in the *tetO-HSP90/hsp90Δ* strain and then subcultured into medium ± 5 μg/mL DOX for 4 hours before protein extraction and western blotting. Protein levels were normalized to the tubulin loading control and quantified compared with the no DOX control. (G) Migration of Rvb1 and Rvb2 upon fluconazole treatment in *S*. *cerevisiae*. Cells were incubated with 0, 4, or 8 μg/mL fluconazole before protein extraction and western blotting with anti-Rvb1 or anti-Rvb2 antibodies. AP-MS, affinity purification-mass spectrometry; BFDR, Bayesian false discovery rate; Casp, caspofungin; DOX, doxycycline; Flu, fluconazole; GFP, green fluorescent protein; SAINTexpress, Significance Analysis of INTeractome; SUMO, small ubiquitin-like modifier.

In addition to the identification of Hsp90 client proteins, our AP-MS experiments uncovered associations between Hsp90 and other chaperone complexes, including the chaperonin containing TCP-1 (CCT) and R2TP complexes (Fig 1). Notably, the physical interactions between Hsp90 and these chaperone complexes were altered upon both antifungal drug treatments (Fig 5A). Additionally, we observed that the Rvb1 protein abundance was decreased in two fractions upon Hsp90 depletion in the SILAC proteomic experiments (S3 Table).

The CCT complex is required for tolerance to caspofungin [27], but the R2TP complex has not been examined in *C*. *albicans*. In *S*. *cerevisiae*, Rvb1 and Rvb2 associate with Hsp90 through the Tah1 and Pih1 co-chaperones, and Hsp90 is required for Pih1 stability [45]. To investigate the relationship between Hsp90 and Rvb1/Rvb2 in *C*. *albicans*, we first examined the levels of Rvb1 and Rvb2 proteins after antifungal drug treatment by western blotting. Upon treatment with fluconazole, the *C*. *albicans* Rvb1 protein migrated as two bands, separated by approximately 10 kDa (Fig 5B). There was no change in the Rvb2 protein migration upon drug treatment (Fig 5B). We then examined Rvb1 levels in response to Hsp90 depletion in the *tetO-HSP90/hsp90*Δ strain. In DOX-treated *tetO-HSP90/hsp90*Δ cells, Rvb1 migrated as two bands, similar to what was seen in response to fluconazole (Fig 5C). DOX treatment of the wild-type strain had no effect on Rvb1 migration (Fig 5C). Overall, this suggests that Hsp90 has a repressive effect on the posttranslational modification of Rvb1.

Next, we explored which posttranslational modification(s) could result in this altered Rvb1 migration. The increase in molecular weight is consistent with sumoylation, and high-throughput studies in *S*. *cerevisiae* predicted an interaction between the small ubiquitin-like modifier (SUMO) Smt3 protein and Rvb1 [46]. To test whether Rvb1 is sumoylated in *C*. *albicans*, we took advantage of an *smt3*Δ*/smt3*Δ mutant strain, in which the only SUMO-encoding locus has been deleted [47]. In the absence of Smt3, treatment with fluconazole did not alter mobility of Rvb1, supporting the hypothesis that the posttranslational modification of Rvb1 is sumoylation (Fig 5D). We then used an anti-Smt3 antibody to immunoprecipitate sumoylated proteins from lysates of the wild-type or *tetO-HSP90/hsp90*Δ strain grown in the presence or absence of DOX. Probing with an anti-Rvb1 antibody revealed a band of the same size as the higher molecular weight Rvb1 band only when Hsp90 was repressed, consistent with Rvb1 being sumoylated in response to Hsp90 depletion (Fig 5E).

One potential mechanism for the increased sumolyation of Rvb1 upon Hsp90 depletion may be through Ulp1, a SUMO deconjugating enzyme that we identified as an Hsp90 physical interactor (Fig 1). If Ulp1 is an Hsp90 client, then compromise of Hsp90 function could reduce SUMO deconjugating activity, thereby increasing Rvb1 sumoylation. Therefore, we examined Ulp1 stability in response to Hsp90 depletion in the *tetO-HSP90/hsp90*Δ strain. In the DOX-treated *tetO-HSP90/hsp90*Δ cells, Ulp1 protein levels were decreased (Fig 5F), suggesting that Ulp1 is a client of Hsp90. Together, these data demonstrate an interaction between Hsp90 and other chaperone complexes, in which Hsp90 represses the sumoylation of Rvb1 through interactions with the client protein Ulp1.

Ulp1 is a known physical interactor of Hsp90 in *S*. *cerevisiae* [46], and the putative sumoylation site in Rvb1 (lysine 228) is conserved between *S*. *cerevisiae* and *C*. *albicans*. Therefore, we examined Rvb1 and Rvb2 levels in *S*. *cerevisiae* after treatment with fluconazole to see whether sumoylation is a conserved fungal response to fluconazole stress. However, we did not observe a shift in molecular weight for either *S*. *cerevisiae* Rvb1 or Rvb2 proteins in response to fluconazole, suggesting that this response may be specific to *C*. *albicans* (Fig 5G).

### Hsp90 stabilizes stress granule and P-body proteins

**Fig 6:**
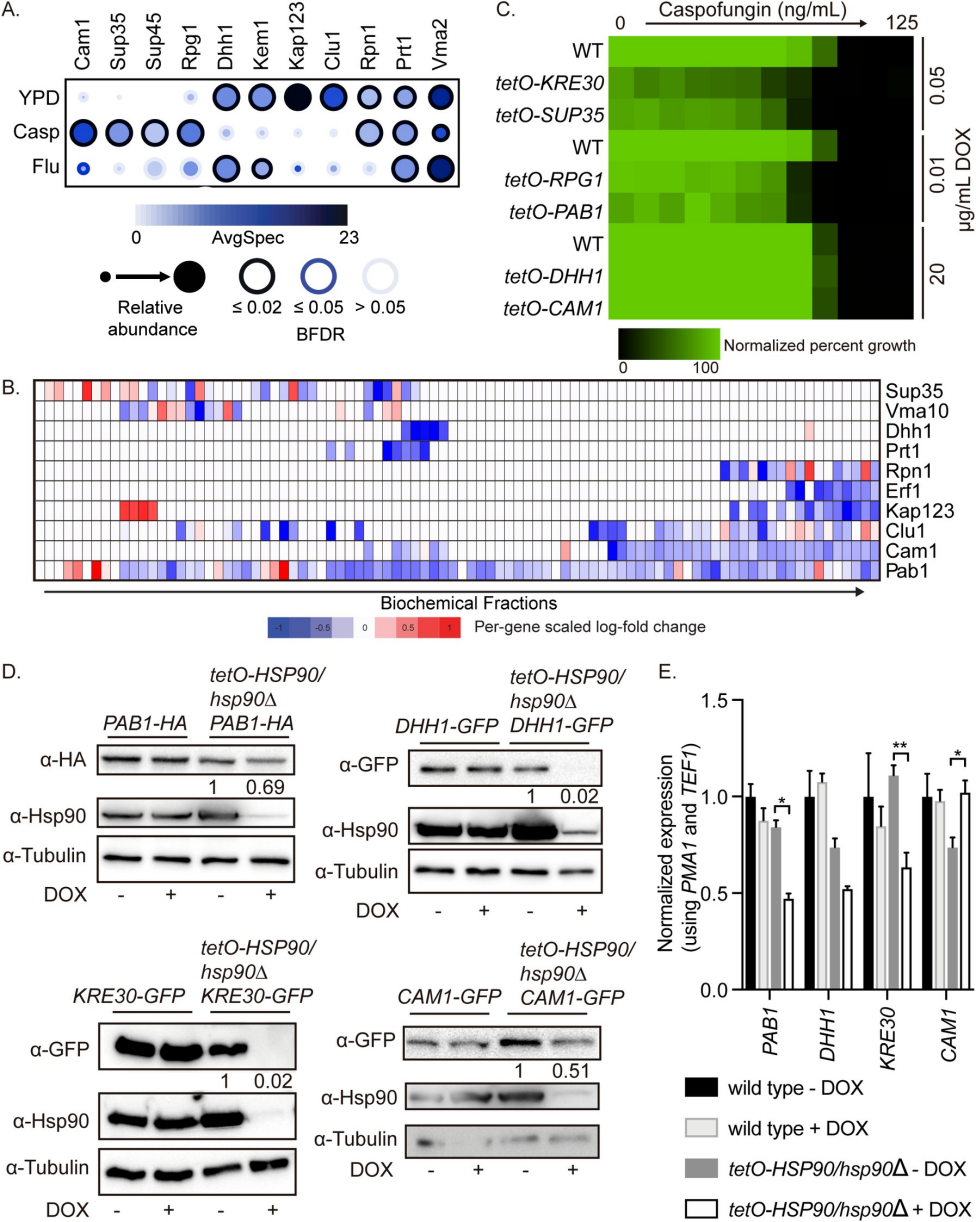
Hsp90 is required for the stability of stress granule and P-body proteins. (A) Stress granule and P-body proteins vary in their interaction with Hsp90 upon drug treatment. AP-MS was performed on *HSP90*^*E36A*^*-GFP/HSP90* cells grown at 30°C in the presence or absence of 8 μg/mL fluconazole (Flu) or 100 nM caspofungin (Casp). Statistically significant Hsp90 interaction partners are shown as a dot plot in which the node color represents the absolute spectral count, node size represents relative abundance between the conditions, and node edge color represents the SAINTexpress BFDR rate. Raw data for this figure can be found in S4 Table. (B) Stress granule protein abundance upon Hsp90 depletion. Protein abundance across 120 IEX fractions in untreated cells or cells treated with DOX to transcriptionally repress *HSP90* in the *tetO-HSP90/hsp90Δ* strain. Raw data for this figure can be found in S3 Table. (C) Stress granule proteins are required for caspofungin tolerance. Mutants of proteins involved in stress granules (Pab1, Kre30, and Sup35) are hypersensitive to caspofungin. MIC assays were performed in YPD medium at 30°C for 24 hours, and optical densities at 600 nm were averaged for two biological replicates performed in technical duplicate. Growth was normalized to the no drug well. To repress target gene expression, the strains were incubated in the indicated concentrations of DOX. Raw data for this figure can be found in S1 Data. (D) Stress granule and P-body protein levels are decreased upon transcriptional repression of *HSP90*. Cells were grown overnight in ±0.5 μg/mL DOX to repress *HSP90* in the *tetO-HSP90/hsp90Δ* strain and then subcultured into medium ± 5 μg/mL DOX for 4 hours before protein extraction and western blotting. Protein levels were normalized to the tubulin loading control and quantified compared with the no DOX control. (E) Stress granule protein transcripts are not dependent on Hsp90. Cells were grown overnight in ±0.5 μg/mL DOX to repress *HSP90* in the *tetO-HSP90/hsp90Δ* strain and then subcultured into medium ± 5 μg/mL DOX for 4 hours before RNA extraction and qRT-PCR. Transcript levels were normalized to *PMA1* and *TEF1*. Significance was determined by one-way ANOVA. ** indicates P value <0.01, * indicates P value <0.05. Raw data for this figure can be found in S1 Data. AP-MS, affinity purification-mass spectrometry; BFDR, Bayesian false discovery rate; DOX, doxycycline; GFP, green fluorescent protein; IEX, ion exchange; MIC, minimum inhibitory concentration; P-body, processing body; qRT-PCR, quantitative reverse transcription PCR; SAINTexpress, Significance Analysis of INTeractome; YPD, yeast extract peptone dextrose.

Through our proteomic experiments, we identified that Hsp90 interacts with or regulates the stability of many proteins involved in P-bodies, stress granules, and RNA binding. In our AP-MS experiments, we identified multiple stress granule or P-body proteins that interact with Hsp90, especially upon treatment with caspofungin (Fig 6A). We also observed that some of these proteins were depleted upon *HSP90* transcriptional repression in our SILAC analysis (Fig 6B). This suggests that these proteins might be clients of Hsp90 in *C*. *albicans* with roles in caspofungin tolerance. Therefore, we tested whether any of these proteins with increased association with Hsp90 during caspofungin stress were required for caspofungin tolerance by measuring susceptibility upon transcriptional repression of the target gene using strains from the gene replacement and conditional expression (GRACE) collection [48,49]. Depletion of Pab1, Sup35, Rpg1, or Kre30 rendered the cells hypersensitive to caspofungin (Fig 6C) but not fluconazole (S4A Fig), demonstrating a specific role for Pab1, Sup35, Rpg1, and Kre30 in caspofungin tolerance.

To test whether some of these proteins with roles in stress granules or P-bodies depend on Hsp90 for stability, we generated C-terminally GFP- or HA-tagged strains in the wild-type and *tetO-HSP90/hsp90*Δ backgrounds. We chose Pab1, as it is a canonical stress granule marker, and Dhh1 as a canonical P-body marker [50,51], as well as Cam1 and Kre30, which are predicted to aggregate in *C*. *albicans* based on similarity to their *S*. *cerevisiae* homologs (Cam1 and Arb1) but have not been characterized previously in *C*. *albicans*. We monitored the stability of each of these proteins via western blotting after transcriptional repression of *HSP90* with DOX. We observed that Kre30 and Dhh1 were highly dependent upon Hsp90 for stability, with the GFP-tagged proteins reduced to close to the detection limit upon Hsp90 depletion (Fig 6D). *KRE30* transcript levels were reduced by 50% upon Hsp90 depletion, while *DHH1* transcripts were unchanged (Fig 6E), demonstrating that the impact of Hsp90 depletion on Kre30 and Dhh1 is largely at the protein level. Cam1 stability was also dependent on Hsp90, with a 50% decrease in protein levels despite an increase in transcript levels upon Hsp90 depletion (Fig 6D and Fig 6E). Finally, Pab1 did not appear to depend on Hsp90 for stability, as there was only a 30% decrease in protein levels and a corresponding 50% decrease in transcript levels upon Hsp90 depletion (Fig 6D and Fig 6E). Together, this suggests that Kre30, Dhh1, and Cam1 are clients of Hsp90, while Pab1 is not. We also examined the levels of Pab1, Cam1, Kre30, and Dhh1 upon fluconazole or caspofungin treatment, and observed no major decreases in protein or transcript levels (S4B and S4C Fig). This suggests that the observed changes in peptide counts for these proteins under drug stress are not due to a lack of the bait protein and instead could reflect a change in localization or requirement for chaperoning by these proteins.

**Fig 7:**
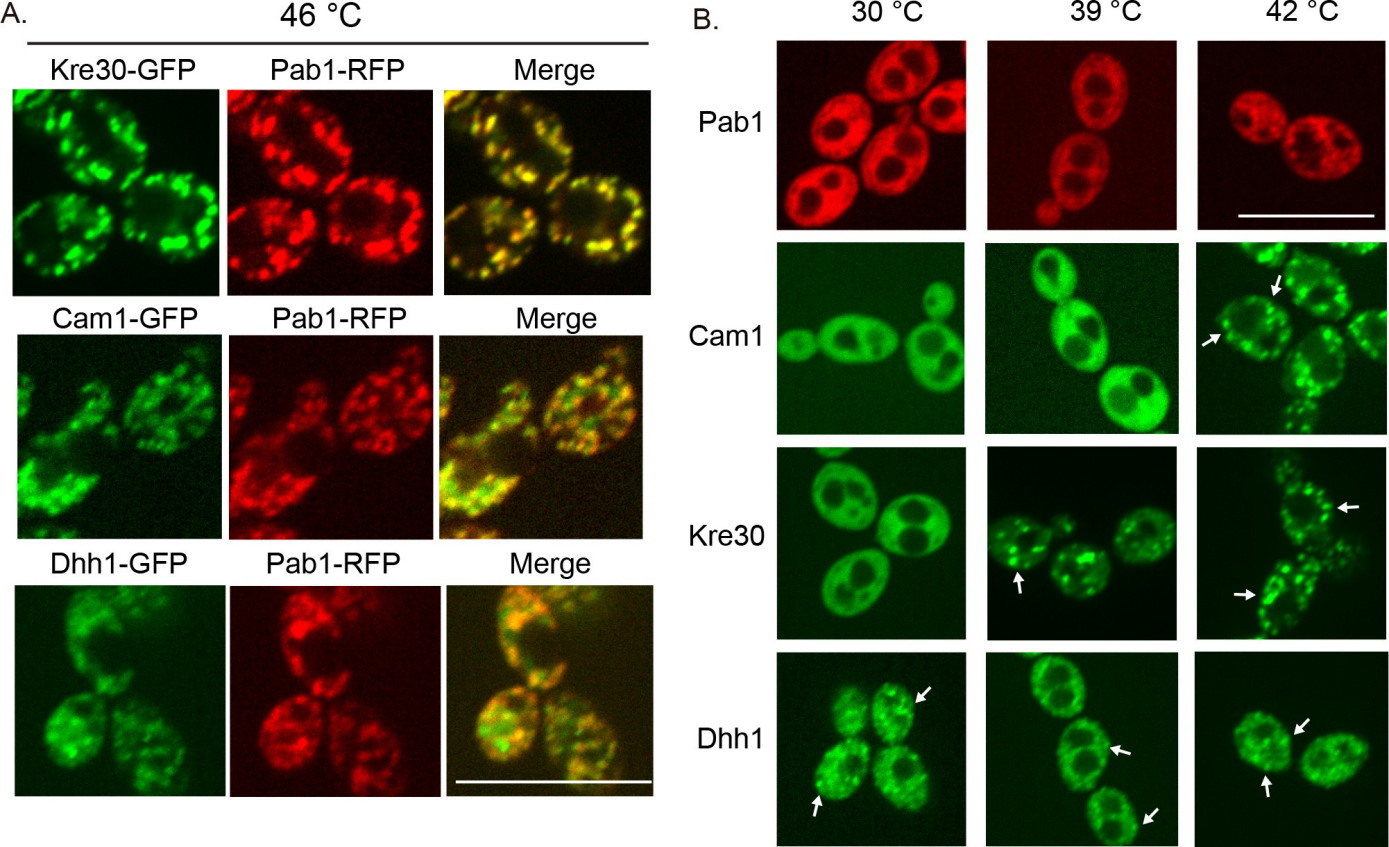
Stress-dependent protein aggregation correlates with dependence on Hsp90 for stability. (A) Identification of stress granules and P-bodies in *C*. *albicans*. Cells were grown overnight in YPD, diluted to an OD_600_ of 0.1, and subcultured for 4 hours before a 10-minute incubation at 46°C. Scale bar is 10 μm. (B) Protein aggregation depends on temperature. Cells were grown overnight in YPD, diluted to an OD_600_ of 0.1, and subcultured for 4 hours at 30°C before a 10-minute incubation at the indicated temperatures. Scale bar is 10 μm. Arrows indicate aggregates. GFP, green fluorescent protein; OD_600_, optical density at wavelength 600 nm; P-body, processing body; RFP, red fluorescent protein; YPD, yeast extract peptone dextrose.

Previous work in mammalian cells demonstrated that Hsp90 inhibition prevents P-body formation and decreases localization of specific protein to stress granules [52–54]. To test whether Hsp90 influences stress granule or P-body formation in *C*. *albicans*, we first needed to establish that these proteins form aggregates, and differentiate between stress granules and P-bodies based on co-localization with the stress granule protein Pab1. We tagged Pab1 with red fluorescent protein (RFP) in the Cam1, Kre30, and Dhh1 GFP-tagged strain backgrounds and then treated the cells with a short heat shock (46°C for 10 minutes), as this treatment is sufficient to induce reversible protein aggregation in *S*. *cerevisiae* [2]. We observed clear punctae of fluorescence in strains with tagged Pab1, Kre30, Cam1, and Dhh1 after a 10-minute incubation at 46°C (Fig 7A), consistent with the formation of stress-induced aggregates. Pab1 appeared to co-localize with Kre30, suggesting that these proteins are primarily in stress granules at 46°C. However, Pab1 did not fully co-localize with Cam1 or Dhh1, consistent with these proteins forming P-bodies that can be separate from the stress granules (Fig 7A).

We then tested a range of temperature treatments to find the minimum temperature required to induce aggregation for each of these proteins. We hypothesized that increasing thermal stress would gradually overwhelm the functional capacity of Hsp90 and prevent it from chaperoning these proteins. The P-body protein Dhh1 was present as punctae at 30°C (Fig 7B), consistent with the idea that P-bodies are present at low numbers under basal growth conditions and increased upon stress [55]. Similar aggregates were observed when Dhh1 was tagged with RFP (S5 Fig). Surprisingly, treatment at a host physiological febrile temperature stress of 39°C for 10 minutes was sufficient to induce aggregates of Kre30 (Fig 7B). Aggregation of Cam1 occurred after incubation at 42°C for 10 minutes (Fig 7B), and Pab1 only formed aggregates after incubation at 46°C for 10 minutes (Fig 7A and Fig 7B). The temperature at which these proteins aggregated is correlated with the dependence of each protein on Hsp90 for stability (Fig 6D), with Dhh1 and Kre30 showing both a strong dependence on Hsp90 for stability and a propensity towards aggregation. Together, this suggests that Hsp90 chaperone function is required to regulate the aggregation and localization of these proteins.

## Discussion

Fungal pathogens can thrive in diverse and hostile environments, at least in part due to a diverse repertoire of stress response pathways that are enabled by the molecular chaperone Hsp90. Here, we report on two complementary large-scale approaches to interrogate the Hsp90 physical interaction network in a leading human fungal pathogen, *C*. *albicans*. The data obtained in this integrative proteomic study allowed us to map the Hsp90 physical interaction network and identify proteins that depend on Hsp90 for stability.

To build a protein–protein interaction network for Hsp90, we performed AP-MS on C-terminally tagged Hsp90 with the E36A “trap” mutant, which allows for the identification of increased numbers of Hps90 client proteins based on this substitution preventing the normal Hsp90 chaperone cycle. A limitation of this dataset is that we focused on interactions obtained with C-terminally tagged Hsp90. The presence of a C-terminal tag does not interfere with Hsp90 dimerization (S1 Fig), but it may prevent the binding of proteins to the C-terminal domain of Hsp90, especially those that bind to the TPR domain [15]. We also leveraged our AP-MS analysis of nine Hsp90 co-chaperones to illuminate additional components of the Hsp90 physical interaction network in *C*. *albicans*.

In parallel, we examined global proteome changes upon Hsp90 perturbations by measuring protein abundance and elution profiles in response to both transcriptional repression and pharmacological inhibition of Hsp90. Similar to our previous RNA sequencing results [56], transcriptional repression and pharmacological inhibition of Hsp90 resulted in different effects to the cell, with Hsp90 transcriptional repression having more dramatic effects on the proteome (S3 Table). Together, our data greatly expand upon what we know about the Hsp90 interactome in this fungal pathogen, as only 25% of the identified interactors had been previously described in other species. This provides a foundation for investigating the specific roles for this conserved chaperone protein in this pathogen.

We leveraged these analyses to uncover new mechanisms through which Hsp90 regulates cellular responses to stress. First, we explored the role of Hsp90 as a classic chaperone of signal transduction cascades. We established that Pbs2, the kinase directly upstream of Hog1, is a client of Hsp90. The Hog1 signalling cascade is a key regulator of virulence, stress tolerance, and antifungal drug resistance [57–59]. Similar to what we have previously observed for the Pkc1-MAPK cascade [27], this analysis suggests that Hsp90 stabilizes multiple components of the Hog1-MAPK cascade [28], although the effects of Hsp90 on allowing the accumulation of the phosphorylated form of Hog1 may be due in part to Hsp90 chaperoning of the Pbs2 kinase. Notably, previous work in *S*. *cerevisiae* demonstrated that multiple components of a signalling cascade may be degraded upon Hsp90 depletion [60]; however, this did not appear to be through direct chaperoning of these proteins. Further analysis of the dynamic interactions between *C*. *albicans* Hsp90 and multiple components of a single signalling cascade will likely reveal new insights into molecular control that enables the rapid sensing of environmental stresses and orchestration of crucial adaptive responses. Potentially, chaperoning multiple components of the same cascade may allow for fine-tuning of stress responses.

We also explored the role of Hsp90 in regulating protein posttranslational modification. The R2TP complex associates with Hsp90 in many organisms [61]. In *C*. *albicans*, we observed a physical interaction between Hsp90 and both Rvb1 and Rvb2, with the strength of the association modulated by stress (Fig 5A). We established that Rvb1 is sumoylated upon depletion of Hsp90 or upon fluconazole-induced cell membrane stress, which is thought to increase the cellular demand for Hsp90 and overwhelm its functional capacity [33]. This specific posttranslational modification of Rvb1 has not been previously explored in other organisms, although in mammalian cells, Rvb2 is sumoylated to control nuclear localization of the protein [62]. Notably, we did not observe a change in either Rvb1 or Rvb2 posttranslational modifications in *S*. *cerevisiae* upon fluconazole treatment, suggesting that this regulation of the R2TP complex by Hsp90 may be specific to *C*. *albicans*. Future experiments will address the functions of the R2TP complex in chromatin remodeling, telomerase assembly, and small nucleolar ribonucleoprotein (snoRNP) synthesis in *C*. *albicans* and how these functions are regulated by Hsp90 [61].

Our findings also implicate Hsp90 in regulating mRNA-associated granule formation upon stress. The canonical chaperones involved in the formation and disaggregation of mRNA-associated granules are Hsp70 and Hsp104 [5]. However, we demonstrate that Hsp90 is required for the stability of multiple predicted mRNA binding proteins, including Dhh1, Kre30, and, Cam1. We established that all of these proteins form aggregates in response to heat stress, but that the minimum temperature required to induce aggregation varied for each protein. The proteins that were most dependent on Hsp90 for stability were also those that aggregated at the lowest temperatures. This suggests that in *C*. *albicans*, Hsp90 may be the key chaperone that regulates aggregation, as deletion of Hsp104 has little effect [63]. This may have profound implications for stress granule assembly in other systems, such as in the context of human cells, where protein interactions in stress granules are modulated by stress and aberrant dynamics are associated with disease [64].

Given the importance of Hsp90 in eukaryotic cell biology, exploring the chaperone network is a powerful approach to probe new facets of cellular function and regulation and to identify novel targets for therapeutic intervention. As a core hub of protein homeostasis and cellular signalling, Hsp90 has emerged as a promising target for diverse diseases, including cancer, neurodegenerative diseases, and infections caused by eukaryotic pathogens [65,66]. As a highly conserved chaperone that is essential for the viability of all eukaryotes tested, there are inherent challenges of selectively targeting Hsp90 in the context of disease while mitigating host toxicity. There has been considerable progress with targeting specific isoforms of Hsp90 [67,68], and there is potential to exploit differences in Hsp90 conformational states between species [69,70]. Our findings further emphasize the opportunity of targeting other components of the Hsp90 chaperone network. We find that there are many Hsp90 clients and functional relationships that are not conserved between *C*. *albicans* and the model yeast. This may suggest differences in biophysical properties of specific proteins between the species that alter dependence on Hsp90 or that organisms can engage distinct strategies to regulate the form and function of metastable proteins. These possibilities resonate with our findings of distinct Hsp90 interaction profiles in response to stress, which can alter protein conformation, activation, localization, and expression levels. By mapping the Hsp90 chaperone network, we have revealed new vulnerabilities in fungal pathogens that could be exploited for the development of strategies to cripple fungal pathogens and treat life-threatening infectious disease.

## Methods and materials

### Strains and culture conditions

All strains were maintained in cryo-culture at –80°C in 25% glycerol and passaged in YPD. For solid medium, 2% agar was used. Strains that were auxotrophic for uridine were grown with 80 mg/L uridine added to the growth medium. When indicated, strains were grown in the presence of DOX (Doxycycline Hydrochloride, DB0889, BioBasic, Markham, ON, Canada) dissolved in water. For experiments to repress gene expression, unless indicated otherwise, overnights of relevant strains and controls were subcultured in the absence or presence of 0.5 μg/mL DOX and grown overnight. The cells were subcultured into 5 μg/mL DOX and grown to mid-log phase for qRT-PCR experiments and western blot analysis.

For heat shock experiments, overnights of relevant strains were grown in YPD at 30°C and then diluted to an OD_600_ of 0.1 and subcultured for 4 hours to mid-log phase before 10 minutes of heat shock at the indicated temperatures.

Individual strains are listed in S5 Table. Plasmids used for strain construction are included in S6 Table. All primer sequences are included in S7 Table.

### Protein extraction for SILAC proteomics

The *lys2Δ/Δ tetO-HSP90/hsp90Δ* strain was incubated overnight, either using unlabelled lysine, heavy lysine (L-Lysine-^13^C_6_,^15^N_2_ hydrochloride), or medium lysine (L-Lysine-4,4,5,5-d_4_ hydrochloride) (Sigma-aldrich, Oakville, ON, Canada). The sample for *HSP90* genetic depletion was treated with 0.05 μg/mL DOX overnight. The next day, the samples were subcultured into YNB with the same lysine at an OD_600_ of 0.1 in 50 mL. For *HSP90* genetic depletion, the sample was treated with 5 μg/mL DOX. For Hsp90 pharmacological inhibition, the sample was treated with 15 μM GdA. Cells were then harvested at an OD_600_ of between 0.6 and 0.8, collected at 3,000 rpm for 10 minutes at 4°C, washed with ice-cold water, and snap frozen in an ethanol-dry ice bath.

Cells were then lysed in a 1:1 volume of lysis buffer (50 mM HEPES, pH 7.4, 1 mM EDTA, 4 mM EGTA, 1 mM Dithiothreitol, 2 mM MgCl_2_, 10% v/v glycerol, 100 units/mL Benzonase nuclease [Sigma-aldrich, Oakville, ON, Canada], 1 × EDTA-free protease inhibitor [Sigma-aldrich, Oakville, ON, Canada]) with glass beads and vortexing for 5–8 minutes at 4°C. The extent of cell lysis was visually controlled by microscope and lysis stopped when 70%–90% of cells were successfully lysed. The samples were then spun to remove the glass beads and the supernatant collected in a fresh tube. The cell debris was removed by further centrifugation for 15 minutes at 14,000 rpm at 4°C. The supernatant was then filtered using a 0.45-μm centrifugal filter device. The lysate was saved, as the protein extract and Bradford assays were performed to determine protein concentrations.

### IEX-HPLC

IEX-HPLC fractionations were performed using an Agilent 1260 infinity HPLC system (Agilent Technologies, Mississauga, ON, Canada) equipped with a mixed-bed PolyCATWAX chromatography column (200 × 2.1 mm i.d., 5 μm, 1,000-Å; PolyLC, MD). IEX buffers were always freshly prepared with HPLC grade H_2_O and comprised of a low salt Buffer A (10 mM MES, pH 6, 0.01%-NaN_3_, 5%-Glycerol) and high salt Buffer B (Buffer A + 1.5 M NaCl). Before separating protein extracts by PolyCATWAX column, the column was conditioned by running two consecutive blank gradients using the same MES buffer system used for protein separations and then equilibrated with Buffer A for 30 minutes. Approximately 1.5 mg of soluble protein extract was loaded onto the column, and bound proteins were eluted using a salt gradient as follows. After 10 minutes isocratic hold at 100% Buffer A, a shallow linear gradient to 15% Buffer B from 10 to 120 minutes was applied, followed by a linear gradient to 50% Buffer B from 120 to 180 minutes and a steep gradient to 100% Buffer B from 180 to 210 minutes. An isocratic hold at 100% Buffer B was applied until 240 minutes to remove highly bound protein complexes. Protein elution was monitored by UV absorbance at 280 nm. The gradient was run at a flow rate of 0.2 mL/minute and 120 fractions were collected.

### Protein precipitation and digestion

HPLC fractions were precipitated overnight at 4°C by adding 10% v/v trichloroacetic acid (TCA). Precipitate was collected at 20,000*g* for 30 minutes and the pellets washed twice with 300 μL ice-cold acetone. The pellets were air-dried and then dissolved in 90 μL 50 mM NH_4_HCO_3_. The samples were reduced by adding DTT (Thermo) to a final concentration of 5 mM and incubated for 20 minutes at 50 ˚C with gentle agitation. The samples were cooled to room temperature and alkylated by adding 10 mM Iodoacetamide (Sigma-aldrich, Oakville, ON, Canada) and incubating in the dark for 20 minutes. To quench excess Iodoacetamide, 5 mM DTT was added to each sample. The protein fractions were then digested by adding 1 μg of mass spectrometry grade trypsin gold (Promega, Madison, WI) and incubated overnight at 37°C with gentle agitation. The digestion was quenched by adding formic acid (FA) to 1% (v/v) final concentration and the peptide mixtures were purified using ziptip C18 tips (Millipore). The ziptip C18 tips were preconditioned with 10 μL acetonitrile, followed by 2 × 10 μL of 0.1% trifluoroacetic acid (TFA). After loading the peptide mixture onto ziptip C18 tips, the samples were washed three times with 0.1 (v/v) TFA and eluted with 2 × 10 μL elution buffer (80% acetonitrile, 0.1% TFA). The eluates were then lyophilized by Speed Vac (Thermo Scientific, Mississauga, ON, Canada) and dissolved in 1% TFA for LC-MS/MS analysis.

### SILAC LC-MS/MS analysis

LC-MS/MS analyses were performed on an EASY nLC 1200 system coupled to a Q Exactive HF mass spectrometer equipped with an EASY-Spray ion source (all from Thermo Scientific, Mississauga, ON, Canada). A C18 Acclaim PepMap 100 pre-column (3 μm, 100 Å, 75 μm × 2 cm) connected to a PepMap RSLC C18 analytical column (2 μm, 100 Å, 75 μm × 50 cm) (all from Thermo Scientific) was used to separate peptide mixtures prior to injection into the mass spectrometer. Sixty-minute gradients were used to elute peptides from columns. The quality of LC-MS/MS analysis was repeatedly controlled for by running trypsin-digested BSA MS Standard (BioLabs) between sample runs. The acquired MS data were searched using MaxQuant version 1.6.0.16 [71] against the UniProt reviewed *C*. *albicans* proteome protein sequence database.

### SILAC data analysis

SILAC intensities by condition (untreated, *HSP90* depletion and Hsp90 inhibition), elution fraction (1–120), and protein were read into R [72,73] and mapped to *C*. *albicans* SC5314 genes downloaded from the Candida Genome Database June 10, 2018 [74], yielding 1,177 unique genes. Intensity in the data is a measure of abundance of that protein in a particular fraction. We then normalized the data for each protein so that the intensities across all fractions are scaled such that the maximum value of the intensities is 1, using the following formula: intensity/max(intensity). For example, if the scaled intensity is 0.5 in a particular fraction, the protein abundance is half the maximum abundance of that protein in the fractions. For pharmacological and genetic depletion conditions, the log-fold change was computed. The normed log-fold change was computed as follows: log(fold-change)/max(log(fold-change)), across all fractions.

Gene order in Fig 2A was computed using the seriation R package [75]. Intensity spark lines and heat-map representations were visualized using the gplots [76] and ggplot2 [73] R packages. Clusters were computed using the affinity propagation R-package [40,77] over Spearman rank correlation coefficient similarities [39] between wild-type elution intensity profiles.

### AP-MS experiments

AP-MS experiments were performed as previously described [78], with minor modifications. Briefly, GFP-tagged Hsp90^E36A^ and TAP-tagged co-chaperone (Cdc37, Cns1, Cpr6, Cpr7, Aha1, Hch1, Sgt1, Sba1, Sti1) strains were grown overnight at 30°C in YPD. Stationary phase cultures were diluted to an OD_600_ of 0.1 in 1 L YPD and grown to an OD_600_ between 0.6 and 0.8. Cells were harvested at 3,000 rpm for 30 minutes at 4°C, washed with ice-cold water, and snap frozen in an ethanol-dry ice bath. As a control, GFP-tagged Eno1 or TAP-tagged Cas5 cells were prepared in the same manner. For drug treatments, GFP-tagged Hsp90^E36A^ cells were grown overnight at 30°C in YPD. Stationary phase cultures were diluted to an OD_600_ of 0.1 in 1 L YPD and grown to an OD_600_ between 0.6 and 0.8. Cells were incubated with 8 μg/mL fluconazole for the entire 4-hour subculture, or 100 nM caspofungin for the last hour of subculture before collection. As a control, GFP-tagged Eno1 cells were prepared in the same manner.

For protein extraction, the samples were diluted 1:1 by weight in lysis buffer (50 mM Na-HEPES [pH 7.5], 150 mM NaCl, 5mM EDTA, 5mM DTT, 0.1% NP-40, 1× ROCHE protease inhibitor cocktail tablet [Roche Diagnostics, Mississauga, ON, Canada]) and vortexed with glass beads (0.5 mm) for 4 × 1 minute, with 1 minute on ice in between to lyse the cells. Lysates were collected by stacked transfer for 1 minute at 1,000 rpm with a 27½-gauge needle and clarified by centrifugation at 14,000 rpm for 20 minutes in 4°C in a microcentrifuge.

For affinity purification of TAP-tagged interacting proteins, the lysate was passed through a 0.45-μm filter and treated with 2.5 μL benzonase for each mL of lysate on ice for 15 minutes. Cross-linked human IgG magnetic beads (Invitrogen, Mississauga, ON, Canada) were washed with lysis buffer and then 10 μL of the original slurry volume was added to the lysate and incubated at 4°C for 2 hours. The beads were then washed with lysis buffer and the beads were collected by magnetic separation. The samples were then resuspended in fresh lysis buffer and washed three times with 0.5 mL TEV buffer without DTT. Then, 100 μL TEV buffer with 5 mM DTT and 2.5 μL TEV was added to the samples and left to react at 4°C overnight. The next day, 5 μL calmodulin beads per sample were washed three times with 0.5 mL binding buffer (10 mM β-ME, 150 mM NaCl, 1 mM MgOAc, 1 mM Imidazole, 0.1% NP-40, 2 mM CaCl_2_, 10 mM Hepes-KOH [pH 8.0]). The TEV-cleaved samples were then added and incubated at 4°C for 2 hours. The beads were then spun and washed three times with rinsing buffer (50 mM ammonium bicarbonate [ABC], 75 mM NaCl, 1 mM MgOAc, 1 mM Imidazole, 2 mM CaCl_2_) and then eluted twice in 5 μL of eluting buffer (50 mM ABC, 25 mM EGTA) at 4°C for 15 minutes. The samples were then digested with 0.5 μg trypsin in 100 mM fresh ABC overnight at 37°C. The next day, the samples were treated with HAc (0.1%) and then used for MS.

To affinity purify the GFP-interacting proteins, we used the GFP trap affinity resin (ChromoTek, Martinsried, Germany). The GFP-Trap resin was equilibrated three times with 1 mL of with lysis buffer (50 mM Hepes-Na [pH 7.5], 150 mM NaCL, 5 mM EDTA, 5 mM DTT, 0.1% NP-40, 1× ROCHE protease inhibitors [Roche Diagnostics, Mississauga, ON, Canada]), using 25 μL resin for each 1-L culture. The protein extract was then added to the resin and rotated for 2 hours at 4°C. The beads were then washed with lysis buffer and then wash buffer. Then, the samples were digested through On-Bead Trypsin-Digestion. Briefly, beads were washed with 20 mM Tris-HCl (pH 8.0) and then incubated with trypsin (0.2 μg/μL in 20 mM Tris-HCl, pH 8.0) at 37°C for 4 hours with rotation. The beads were then magnetically removed and the supernatant was transferred to a fresh tube and incubated with trypsin without rotation at 37°C overnight. The next morning, acetic acid was added to a final concentration of 1%–2% and the samples were kept at −80°C until used for MS.

AP-MS samples and controls were analyzed by MS in two biological replicates, as previously described [78]. MS data generated were stored, searched, and analyzed using the ProHits laboratory information management system (LIMS) platform [79] and searched using Mascot (v2.3.02) and Comet (v2012.02 rev.0) against the *C*. *albicans* RefSeq database (version 68), as previously described [78]. The database parameters were set to search for tryptic cleavages, allowing up to two missed cleavage sites per peptide, with a mass tolerance of 35 or 40 ppm for precursors with charges of 2+ to 4+ and a tolerance of ± 0.15 amu for fragment ions. Variable modifications were deamidated asparagine and glutamine residues and oxidized methionine residues. The results from each search engine were analyzed through the Trans-Proteomic Pipeline (TPP v4.6 OCCUPY rev 3) [80] via the iProphet pipeline [81]. SAINTexpress (v3.3) [82] was used as a statistical tool to calculate the probability value of each potential protein–protein interaction from background contaminants using default parameters and a ProteinProphet cutoff of 0.95. Controls were kept uncompressed and a BFDR of 2% or lower was required for proteins to be classified as significant interaction partners.

### AP-MS data visualization

Dot plots and a heat map of interaction networks obtained by AP-MS were generated using a web-based tool [83]. AP-MS results were loaded into R and mapped to *C*. *albicans* SC5314 genes downloaded from the Candida Genome Database June 10, 2018 [74]. *S*. *cerevisiae* cytoplasmic stress granule annotations (GO:0010494) or P-body (GO:0000932) annotations were collected from the *Saccharomyces* Genome Database (SGD) database in April 2019 [84]. *S*. *cerevisiae* physical interactions were collected from BioGRID [85]. Cytoscape 3.6.1 [86] was used to construct Fig 1.

### Protein extractions and western blotting

Strains were inoculated overnight in YPD, subcultured to an OD_600_ of 0.1 in 10 mL of fresh YPD, incubated for 4 hours at 30°C with shaking, and then collected. To repress *HSP90* expression, cells were grown overnight in ±0.5 μg/mL DOX and then subcultured at a starting OD_600_ of 0.3 into media ±5 μg/mL DOX before collection. For drug treatments, cells were incubated with 8 μg/mL fluconazole for the entire 4-hour subculture, or 100 nM caspofungin for the last 1 hour of subculture before collection. Pellets were washed once in 1× PBS before freezing at −80°C. The pellets were then resuspended in lysis buffer (50 mM HEPES, pH 7.4, 150 mM NaCl, 5 mM EDTA, 1% Triton-X100, 100 mM NaF, 20 mM Na_3_VO_4_, 1 mM PMSF, and protease inhibitor cocktail complete, EDTA-free tablet, [Roche Diagnostics, Mississauga, ON, Canada]) and lysed by bead beating. Protein levels were determined by Bradford assays, normalized to 2 mg/mL, and boiled in 6× Laemmli buffer for 3 minutes before loading on 8% SDS-PAGE gels.

For all westerns, separated proteins were electrotransferred to polyvinylidene difluoride (PVDF) membrane (Bio-Rad Laboratories, Mississauga, ON, Canada) for 90 minutes. Blots were blocked with 5% skim milk in phosphate-buffered saline with 0.1% Tween 20 (PBS-T). The molecular weights were determined by electrophoresis of marker proteins in the PageRuler pre-stained protein ladder (Invitrogen, Mississauga, ON, Canada).

HA epitopes were detected using a 1:5,000 dilution of anti-HA primary antibody (1:2,500, Cedarlane Clone: HA.C5, antibody ID, AB_10278484, Burlington, ON, Canada). GFP epitopes were detected using an anti-GFP antibody (1:5,000, Chromotek 3H9, antibody ID, AB_10773374, Martinsried, Germany). Hsp90 was detected using an anti-Hsp90 antibody (1:10,000, gift from B. Larson). Rvb1 was detected using an anti-Rvb1 antibody (1:2,000, gift from W. Houry). Rvb2 was detected using an anti-Rvb2 antibody (1:2,000, gift from W. Houry). Tubulin was detected using an anti-Tubulin antibody (1:5,000, AbDseroTec, MCA78G, antibody ID, AB_325005, Hercules, CA). Histone H3 was detected using an anti-H3 antibody (1:5,000, Abcam 1791, antibody ID, AB_302613, Burlingame, CA). Proteins in the proteasome were detected using an antibody specific to a conserved peptide in the 20S alpha proteasome proteins Pre1, Pre2, Pre3, Pre5, Pre6, Pre7 (1:3000, anti-proteasome 20S alpha 1+2+3+5+6+7+8 antibody, Abcam, antibody ID, AB_2171376, Cambridge, United Kingdom). Blots were washed with PBS-T or TBS-T and incubated with FITC-conjugated secondary antibodies diluted 1:5,000 in the block solution. Blots were washed with PBS-T or TBS-T before detecting the signals using an ECL western blotting kit as per the manufacturer’s instructions (Pierce, Mississauga, ON, Canada).

Protein levels were normalized using Tubulin or Histone H3 as loading controls. Protein level quantifications were performed using Fiji [87], normalizing against the loading control, and relative levels were determined by comparison with the no DOX, no drug, or DMSO vehicle control under the same conditions. All blots were performed in at least biological duplicate.

### Immunoprecipitation

Immunoprecipitation experiments were performed as previously described, with minor modifications [88]. Briefly, 50 mL of YPD were inoculated at an OD_600_ of 0.1 from overnight cultures of *C*. *albicans*. For repression of Hsp90 gene expression, identical overnight cultures were grown, supplemented with 0.5 μg/mL DOX, and 50-mL subcultures with 5 μg/mL DOX. All cultures were grown for 4 hours at 30°C in 250 mL Erlenmeyer flasks with 200 rpm shaking to mid-log phase. Cells were collected by centrifugation at 3000*g*, washed in 1 mL ice -cold PBS, collected at 12,000*g* in 2-mL screw-cap tubes, then flash frozen and stored at −80°C.

Cell pellets were then resuspended in 1 mL of lysis buffer containing 20 mM Tris, pH 7.5, 100 mM KCl, 5 mM MgCl, and 20% glycerol, with one protease inhibitor cocktail (complete, EDTA-free tablet, Roche Diagnostics) per 10 mL, 1 mM PMSF (EMD Millipore Chemicals, Etobicoke, ON, Canada), and 20 mM sodium molybdate (Sigma-Aldrich, Oakville, ON, Canada) added fresh before use. The tube was filled with acid-washed glass beads until the beads were just below the meniscus at the top of the tube to reduce foaming during bead beating. Cells were disrupted by bead beating twice for 4 minutes, with a 7-minute break on ice between cycles. Lysates were recovered by a stacked transfer and then clarified by centrifugation at 15,339 rcf for 10 minutes at 4°C. Protein concentrations were determined by Bradford analysis.

Anti-GFP immunoprecipitations were performed with GFP-trap magnetic beads. For each reaction, 50 μL of GFP-Trap magnetic agarose bead slurry (ChromoTek, Martinsried, Germany) were washed 3× in 1 mL ice-cold wash buffer (10 mM TrisHCl, 150 mM NaCl, 0.5 mM EDTA, pH 7.5) and then resuspended in 50 μL wash buffer. The beads were collected by magnetic separation between each wash step. Three hundred microliters of normalized lysates were then diluted in 450 μL ice-cold wash buffer, and 50 μL of washed beads were added. Reactions were incubated with end-over-end rotation for 1 hour at 4°C. Fifty microliters of the samples were removed for analysis of total protein in western immunoblots. The remaining beads were washed 3× in 1 mL ice-cold wash buffer. The washed beads were resuspended in 100 μL of SDS-PAGE loading buffer, incubated at 95°C for 10 minutes, and then analyzed by western immunoblotting on SDS-PAGE gels.

Anti-HA immunoprecipitations were performed with an anti-HA agarose slurry (Pierce, Mississauga, ON, Canada). For each reaction, lysates were incubated with 20 μL of anti-HA agarose slurry, rotating overnight at 4°C. Unbound material was removed by washing five times with 1 mL of tris-buffered saline containing 0.05% Tween-20 (TBST), pulse centrifuging for 10 seconds. Proteins were eluted by boiling in 50 μL of 2× sample buffer. For comparison, input samples were diluted to 50 ng of protein. Samples were then analyzed by western immunoblotting on SDS-PAGE gels.

Immunoprecipitation for Rvb1 using Rvb1, and Smt3, antibodies was as follows:

Antibodies for immunoprecipitation of SUMOylated proteins was the polyclonal rabbit anti-Smt3 (antibody ID, AB_1857652, Sigma-Aldrich, Oakville, ON, Canada). This antibody was cross-linked to Dynabeads Protein G (Invitrogen, Mississauga, ON, Canada) as follows. For each antibody, 200 μL of Dynabead Protein G slurry were washed 3× in 1 mL PBST and then resuspended in 1 mL PBST. The beads were collected by magnetic separation between each wash step. Twenty microliters of antibody stocks were added to the Dynabead suspension and then incubated with end-over-end rotation for 30 minutes at room temperature. The antibody-Dynabead complexes were washed 2× in 1 mL conjugation buffer (20 mM sodium phosphate, 150 mM NaCl, pH 7.5) and then resuspended in 1 mL of conjugation buffer supplemented with freshly prepared 5 mM bis(sulfosuccinimidyl)suberate to cross-link the antibodies to the beads. The reaction was incubated with end-over-end rotation at room temperature for 30 minutes and then quenched by adding 50 μL of 1 M TrisHCl, pH 7.5, and continuing incubation for 15 minutes. The beads were then washed 3× in 1 mL PBST and then resuspended in 200 μL PBST for use in immunoprecipitations.

Three hundred microliters of normalized lysates were diluted by addition of 450 μL ice-cold wash buffer (10 mM TrisHCl, 150 mM NaCl, 0.5 mM EDTA, pH 7.5), and then 50 μL of antibody-conjugated Dynabead suspension were added. Reactions were incubated with end-over-end rotation for 1 hour at 4°C. Fifty microliters of the samples were removed for analysis of total protein in western immunoblots. The remaining beads were washed 3× in 1 mL ice-cold wash buffer. The beads were resuspended in 100 μL of SDS-PAGE loading buffer without β-mercaptoethanol and then incubated at 95°C for 10 minutes. The beads were collected by centrifugation at 12,000*g* and discarded. The supernatant was retained, supplemented with 5 μL β-mercaptoethanol, and then analyzed by western immunoblotting.

### qRT-PCR

Gene expression changes were monitored as previously described [31]. Briefly, strains were subcultured to mid-log phase, pelleted at 3,000 rpm at 4°C, washed once, and snap frozen. Cells were lysed by bead beating, RNA was extracted using the QIAGEN RNeasy kit, and DNase treated using the QIAGEN RNase free DNAase Set, and cDNA was amplified by using the AffinityScript Multi Temperature cDNA Synthesis Kit (Agilent Technologies, Mississauga, ON, Canada) with the provided random primers.

qRT-PCR was performed using a 384-well plate, with a 10 μL reaction volume using Fast SYBR Green Master Mix (Applied Biosystems) and the BioRad CFX-384 Real Time System with the following cycling conditions: 95°C for 3 minutes, then 95°C for 10 seconds and 60°C for 30 seconds, for 40 cycles. The melt curve was completed with the following cycle conditions: 95°C for 10 seconds and 65°C for 10 seconds, with an increase of 0.5°C per cycle up to 95°C. Reactions were performed in triplicate for two biological replicates. All data were normalized to the *PMA1* and *TEF1* reference genes for *C*. *albicans*. Additional gene primer pairs are included in S7 Table. Data were analyzed using the BioRad CFX Manager 3.1. Error bars depict standard error of the means of technical triplicates.

### MIC assays

Drug tolerance assays were performed in flat-bottom, 96-well microtiter plates (Sarstedt, St. Leonard, QC, Canada) using a modified broth microdilution protocol, as previously described [30]. For target gene depletion in the *tetO* strains, cells were incubated overnight without DOX before being assayed for drug sensitivity in the presence of DOX, except for the strains in which 20 μg/mL DOX was used. For these strains, cells were incubated overnight in DOX before being assayed for drug sensitivity in the presence of DOX. Gene depletion at the given concentration of DOX was confirmed by qRT-PCR for each strain. MIC tests were set up in a total volume of 0.2 mL/well with 2-fold dilutions of each drug in YPD, as indicated. Plates were incubated in the dark at 30°C for 24 hours before OD_600_ was determined using a spectrophotometer (Molecular Devices). Each strain was tested in technical and biological duplicates. MIC data were quantitatively displayed with color using Java TreeView 1.1.1 (http://jtreeview.sourceforge.net).

### Microscopy

Fluorescent proteins were visualized with a CSU-X1 spinning disk confocal on a Nikon Eclipse Ti inverted microscope with an Andora Clara digital camera using CFI APO 100× oil TIRF objective. Images were collected using differential interference contrast (DIC), 488-nm excitation (GFP), and 561-nm excitation (RFP) and analyzed with NIS-Elements software 4.10 (Nikon) and Fiji [87].

### Strain construction

Tagged strains were created using a PCR-based strategy [89]. Transformations using nourseothricin (NAT) as the selectable marker were plated on YPD plates containing 150 μg/mL NAT. Transformations using arginine (ARG) or histidine (HIS) as the selectable marker were plated on synthetic defined medium plates that lack that amino acid.

To generate an E36A mutation in one *HSP90* allele (CaLC3104 and CaLC3105), the CaLC239 strain was transformed with pLC759 digested with BssHI and selected for NAT resistance. Upstream integration was tested with oLC275 and oLC276, and downstream integration with oLC274 and oLC335. The genomic DNA was PCR amplified using oLC203 and oLC198 and oLC275 and oLC198, and the PCR product was sequenced with oLC199. This is the CaLC3104 strain. The NAT marker was then flipped on YNB-BSA to restore NAT sensitivity. This is the CaLC3105 strain.

To C-terminally TAP tag the *HSP90*^*E36A*^ allele (CaLC3136), The *TAP-HIS1* cassette was PCR amplified from pLC572 using primers oLC1974 and oLC1975 and transformed into the CaLC3104 strain containing the *HSP90*^*E36A*^ allele. Integration of the *TAP-HIS* cassette was tested using oLC1645 and oLC203 to confirm that the mutant allele was tagged. The NAT marker was then flipped on YNB-BSA to restore NAT sensitivity. Expression of the tagged protein of the right size was confirmed by western blotting.

To C-terminally GFP-tag the *HSP90*^*E36A*^ allele (CaLC3953), the *GFP-NAT* cassette was PCR amplified from pLC389 with oLC1616 and oLC1617. Integration was tested with oLC1976 and oLC2318 and oLC601 and oLC271. Strains with positive integration were amplified with oLC320 and oLC600, and the presence of the E36A mutation was confirmed by sequencing with oLC199. Expression of the tagged protein of the right size was confirmed by western blotting and microscopy.

To N-terminally GFP-tag Hsp90 (CaLC6029), a *GFP-HSP90-NAT* cassette was designed to avoid disrupting the *HSP90* promoter region. DNA fragments were amplified with 25-bp overhangs to fuse together using NEBuilder HiFi DNA Assembly Kit (New England Biolabs, Whitby, ON, Canada). The *GFP* fragment was amplified from pLC1046 using oLC7584 and oLC1793. The wild-type *HSP90* open reading frame and 300 bp of the terminator region were amplified from pLC811 using oLC7677 and oLC7583. The *NAT* fragment was amplified from pLC49 using oLC3667 and oLC7676. Then, 0.2 pmol of each PCR product was used in the NEBuilder reaction, 20 μL of which were then used as a template for amplification with oLC7580 and oLC7585 to generate *HSP90* homology tails. This construct was transformed into CaLC239. Upstream integration was verified with oLC276 and oLC600, and downstream integration was verified with oLC274 and oLC277. GFP-Hsp90 expression was confirmed by microscopy and western blotting. The strain is NAT resistant.

To N-terminally GFP-tag the *HSP90*^*E36A*^ allele (CaLC6031), a *GFP-HSP90*^*E36A*^*-NAT* cassette was designed to avoid disrupting the *HSP90* promoter region. DNA fragments were amplified with 25-bp overhangs to fuse together using NEBuilder HiFi DNA Assembly Kit (New England Biolabs, Whitby, ON, Canada). The GFP fragment was amplified from pLC1046 using oLC7584 and oLC1793. The *HSP90*^*E36A*^ allele open reading frame and 300 bp of the terminator region were amplified from pLC759 using oLC7677 and oLC7583. The *NAT* fragment was amplified from pLC49 using oLC3667 and oLC7676. Then, 0.2 pmol of each PCR product was used in the NEBuilder reaction, 20 μL of which were then used as a template for amplification with oLC7580 and oLC7585 to generate *HSP90* homology tails for homologous recombination. This construct was transformed into CaLC239. Upstream integration was verified with oLC276 and oLC600, and downstream integration was verified with oLC274 and oLC277. GFP-Hsp90 expression was confirmed by microscopy and western blotting.

To generate an *HSP90/hsp90* heterozygous deletion strain (CaLC627), plasmid pLC62 was digested with KpnI and SacII and transformed into CaLC239. Upstream integration was tested with oLC276 and oLC275, and downstream integration was tested with oLC277 and oLC274. The NAT marker was then flipped out by growing on YNB-BSA to restore NAT sensitivity. Presence of the deleted allele was verified using oLC276 and oLC277.

The *AHA1-TAP-HIS/AHA1-TAP-ARG* strain (CaLC3051) was made by amplifying the *TAP-HIS* cassette from pLC572 and the *TAP-ARG* cassette from pLC573 using oLC2675 and oLC2676. The PCR products were sequentially transformed into CaLC239 background using the auxotrophic markers. Integration of the *HIS* cassette was tested using oLC1635 with oLC2677 and oLC1645 with oLC2678, and integration of the ARG cassette was tested using oLC1593 with oLC2677 and oLC1594 with oLC2678. Expression of the tagged protein of the right size was confirmed by western blotting.

The *SBA1-TAP-HIS/SBA1-TAP-ARG* strain (CaLC3080) was made by amplifying the *TAP-HIS* cassette from pLC572 using oLC2697 and oLC2698 and the *TAP-ARG* cassette from pLC573 using oLC2697 and oLC2698. The PCR products were sequentially transformed into CaLC239 background using the auxotrophic markers. Integration of the HIS cassette was tested using oLC1635 and oLC2699 (upstream) and oLC1645 and oLC2700 (downstream). Integration of the *ARG* cassette was tested using oLC1593 and oLC2699 (upstream) and oLC1594 and oLC2700 (downstream). Expression of the tagged protein of the right size was confirmed by western blotting.

The *SGT1-TAP-HIS/SGT1-TAP-ARG* strain (CaLC3081) was made by amplifying the *TAP-HIS* cassette from pLC572 using oLC2243 and oLC2244, and the *TAP-ARG* cassette from pLC573 using oLC2243 and oLC2244. The PCR products were sequentially transformed into CaLC239 background using the auxotrophic markers. Integration of the *ARG* cassette was tested using oLC1593 and oLC2245 (upstream) and oLC1594 and oLC2246 (downstream). Integration of the *HIS* cassette was tested using oLC1634 and oLC2245 (upstream) and oLC1645 and oLC2246 (downstream). Expression of the tagged protein of the right size was confirmed by western blotting.

The *CDC37-TAP-HIS/CDC37-TAP-ARG* strain (CaLC3083) was made by amplifying the *TAP-HIS* cassette from pLC573 using oLC1699 and oLC1700, and the TAP-ARG cassette from pLC572 using oLC1699 and oLC1700. The PCR products were sequentially transformed into the CaLC239 background using auxotrophic markers. Integration of the *HIS* cassette was tested using oLC1634 and oLC1701 (upstream) and oLC1645 and oLC1702 (downstream). Integration of the *ARG* cassette was tested using oLC1591 and oLC1701 (upstream) and oLC1593 and oLC1702 (downstream). Expression of the tagged protein of the right size was confirmed by western blotting.

The *CPR6-TAP-HIS/CPR6-TAP-ARG* strain (CaLC3084) was made by amplifying the *TAP-HIS* cassette from pLC572 using oLC2683 and oLC2684 and the *TAP-ARG* cassette from pLC573 using oLC2683 and oLC2684. The PCR products were sequentially transformed into the CaLC239 background using auxotrophic markers. Integration of the *HIS* cassette was tested using oLC1634 and oLC2685(upstream) and oLC1645 and oLC2686 (downstream). Integration of the *ARG* cassette was tested using oLC1591 and oLC2685 (upstream) and oLC1593 and oLC2686 (downstream). Expression of the tagged protein of the right size was confirmed by western blotting.

The *CPR7-TAP-HIS/CPR7-TAP-ARG* strain (CaLC3086) was made by amplifying the *TAP-HIS* cassette from pLC572 and the *TAP-ARG* cassette from pLC573 using oLC2687 and oLC2688. The PCR products were sequentially transformed into the CaLC239 background using auxotrophic markers. Integration of the *HIS* cassette was tested using oLC1634 and oLC2689 (upstream) and oLC1645 and oLC2690 (downstream). Integration of the *ARG* cassette was tested using oLC1591 and oLC2689 (upstream) and oLC1593 and oLC2690 (downstream). Expression of the tagged protein of the right size was confirmed by western blotting.

The *CNS1-TAP-HIS/CNS1-TAP-ARG* strain (CaLC3238) was made by amplifying the *TAP-HIS* cassette from pLC572 and the *TAP-ARG* cassette from pLC573 using oLC2713 and oLC2679. The PCR products were sequentially transformed into the CaLC239 background using auxotrophic markers. Integration of the *HIS* cassette was tested using oLC1634 and oLC2681 (upstream) and oLC1645 and oLC2682 (downstream). Integration of the *ARG* cassette was tested using oLC1591 and oLC2681 (upstream) and oLC1593 and oLC2682 (downstream). Expression of the tagged protein of the right size was confirmed by western blotting.

The *STI1-TAP-HIS/STI1-TAP-ARG* strain (CaLC3246) was made by amplifying the *TAP-HIS* cassette from pLC572 and the *TAP-ARG* cassette from pLC573 using oLC2714 and oLC2701. The PCR products were sequentially transformed into the CaLC239 background using auxotrophic markers. Integration of the *HIS* cassette was tested using oLC1634 and oLC2703 (upstream) and oLC1645 and oLC2704 (downstream). Integration of the *ARG* cassette was tested using oLC1591 and oLC2703 (upstream) and oLC1593 and oLC2704 (downstream). Expression of the tagged protein of the right size was confirmed by western blotting.

The *HCH1-TAP-HIS/HCH1-TAP-ARG* strain (CaLC3247) was made by amplifying the *TAP-HIS* cassette from pLC572 and the *TAP-ARG* cassette from pLC573 using oLC3090 and oLC3091. The PCR products were sequentially transformed into the CaLC239 background using auxotrophic markers. Integration of the *HIS* cassette was tested using oLC1634 and oLC3088 (upstream) and oLC1645 and oLC3089 (downstream). Integration of the *ARG* cassette was tested using oLC1591 and oLC3088 (upstream) and oLC1593 and oLC3089 (downstream). Expression of the tagged protein of the right size was confirmed by western blotting.

To construct a TAP-tagged Cas5 strain (CaLC2348), pLC573 was amplified with oLC2272 and oLC2162 and transformed into CaLC239. Upstream integration was verified using oLC2047 and oLC1593. Downstream integration was verified using oLC1594 and oLC2035.

To make a lysine auxotrophic strain (CaLC3327), a NAT-marked deletion cassette was PCR amplified from pLC49 using primers oLC3193 and oLC3194. Downstream integration was tested with oLC274 and oLC3195 and upstream integration was tested with oLC275 and oLC3196. The NAT marker was then flipped in YNB-BSA, and presence of the deletion allele was tested using oLC3195 and oLC3196. The second copy of the *LYS2* gene was then replaced using the same strategy.

To make CaLC3436, which is the tetracycline-repressible *HSP90* in the lysine auxotrophic strain, CaLC3327 was transformed with DNA from pLC62 that was digested with KpnI-HF and SacII overnight to liberate the *HSP90* NAT-marked deletion cassette. Strains were tested for upstream integration using oLC275 and oLC276 and downstream integration oLC277 and oLC274. The strain was flipped in YNB-BSA to restore NAT sensitivity. The remaining allele of *HSP90* was placed under control of the tetracycline-repressible promoter by transforming in the tetO promoter replacement construct, which was amplified from pLC605 using oLC3390 and oLC3220. Upstream integration was tested using oLC294 and oLC534. Downstream integration was tested using oLC300 and oLC408. Absence of a wild-type allele was tested using oLC294 and oLC297. The strain was then flipped in YNB-BSA to restore NAT sensitivity. Filamentation and DOX responsiveness was confirmed using 0.05 μg/mL DOX.

To make the Pbs2-HA tagged strains (CaLC4288, CaLC4289, CaLC4609), the *PBS2-HA-ARG* cassette was amplified from pLC575 using oLC3835 and oLC3836 and transformed into CaLC239, CaLC3385, or CaLC3136 (which are auxotrophic for ARG and HIS) and selected on Synthetic Defined plates supplemented with HIS at 20 mg/L. Downstream integration was tested using oLC3837 and oLC2029 and upstream integration was tested with oLC1594 and oLC3838. Expression of the tagged protein of the right size was confirmed by western blotting.

To make the doubly-tagged Pab1-HA tagged strains (CaLC5302 and CaLC5303), the *PAB1-HA-HIS1* cassette with homology to the *PAB1* locus was PCR amplified from pLC576 using oLC6078 and oLC6079. A transient CRISPR strategy adapted from Min and colleagues [90] was used to generate this strain. The *CaCAS9* cassette was amplified from pLC963 using oLC5974 and oLC5976. The sgRNA fusion cassette was made by PCR amplifying from pLC963 using oLC5978 and oLC6076 (fragment A) and using oLC6077 and oLC5980 (fragment B). Fusion PCR was performed using the nested primers oLC5979 and oLC5981. All PCRs were cleaned using the PCR cleanup kit (QiaQuick, Invitrogen, Mississauga, ON, Canada) and transformed into CaLC239 or CaLC3385 (which are auxotrophic for ARG and HIS) and plated on Synthetic Defined plates supplemented with ARG at 50 mg/L. Upstream integration of the *HA* tag was confirmed using oLC6082 and oLC2029 and downstream integration with oLC1645 and oLC6083. Absence of the wild-type allele was tested with oLC6082 and oLC6083. Expression of the tagged protein of the right size was confirmed by western blotting.

To make the doubly-tagged Dhh1-GFP tagged strains (CaLC5320 and CaLC5332), the *DHH1-GFP-NAT* cassette with homology to the *DHH1* locus was amplified from pLC1046 using oLC6494 and oLC6495. A transient CRISPR strategy was used to generate this strain. The *CaCAS9* cassette was amplified from pLC963 using oLC5974 and oLC5976. The sgRNA fusion cassette was made by PCR amplifying from pLC963 with oLC5978 and oLC6496 (fragment A) and oLC5980 and oLC6497 (fragment B), and then fusion PCR was performed on the fragments using the nested primers oLC5979 and oLC5981. The *GFP-NAT* cassette, sgRNA, and *CAS9* DNA were transformed into CaLC239 or CaLC3385. Upstream integration was tested using oLC600 and oLC6526 and downstream integration was tested using oLC601 and oLC6527. The strain was then flipped on YNB-BSA to restore NAT sensitivity. Absence of the wild-type allele was tested with oLC6526 and oCL6527. Expression of the tagged protein of the right size was confirmed by western blotting and microscopy.

To make the doubly-tagged Cam1-GFP tagged strains (CaLC5262 and CaLC5261), the *CAM1-GFP-NAT* cassette with homology to the *CAM1* locus was amplified from pLC1046 using oLC6012 and oLC6013. A transient CRISPR strategy was used to generate this strain. The *CaCAS9* cassette was amplified from pLC963 using oLC5974 and oLC5976. The sgRNA fusion cassette was made by PCR amplifying from pLC963 with oLC5978 and oLC6011 (fragment A) and oLC5980 and oLC6010 (fragment B), and then fusion PCR was performed on the fragments using the nested primers oLC5979 and oLC5981. The *GFP-NAT* cassette, sgRNA, and *CAS9* DNA were transformed into CaLC239 or CaLC3385. Upstream integration was tested using oLC600 and oLC6149 and downstream integration was tested using oLC601 and oLC6014. Absence of the wild-type allele was tested with oLC6149 and oLC6014. The strain was then flipped on YNB-BSA to restore NAT sensitivity. Expression of the tagged protein of the right size was confirmed by western blotting and microscopy.

To make the doubly-tagged Kre30-GFP tagged strains (CaLC5260 and CaLC5259), the *KRE30-GFP-NAT* cassette with homology to the *KRE30* locus was amplified from pLC1046 using oLC6107 and oLC6108. A transient CRISPR strategy was used to generate this strain. The *CaCAS9* cassette was amplified from pLC963 using oLC5974 and oLC5976. The sgRNA fusion cassette was made by PCR amplifying from pLC963 with oLC5978 and oLC6015 (fragment A) and oLC5980 and oLC6016 (fragment B), and then fusion PCR was performed on the fragments using the nested primers oLC5979 and oLC5981. The *GFP-NAT* cassette, sgRNA, and *CAS9* DNA were transformed into CaLC239 or CaLC3385. Upstream integration was tested using oLC600 and oLC6109 and downstream integration was tested using oLC274 and oLC6110. Absence of the wild-type allele was tested with oLC6109 and oLC6110. The strain was then flipped on YNB-BSA to restore NAT sensitivity. Expression of the tagged protein of the right size was confirmed by western blotting and microscopy.

The singly-tagged Pab1-RFP tagged strains (CaLC5399, CaLC5395, CaLC5397, CaLC4491) were made by amplifying the C-terminal *RFP*-*NAT* cassette from pLC447 using oLC4432 and oLC4433. This construct was transformed into the *DHH1*-*GFP* (CaLC5320), *KRE30-GFP* (CaLC5260), *CAM1-GFP* (CaLC5262), and wild-type (CaLC239) strain backgrounds. NAT-resistant colonies were tested for upstream integration with oLC4434 and oLC4417, and downstream integration with oLC601 and oLC4435. Expression of the tagged protein of the right size was confirmed by western blotting and microscopy.

The doubly-tagged Dhh1-RFP tagged strain (CaLC5488) was made by amplifying the C-terminal *RFP-NAT* cassette from pLC1047 using oLC6806 and oLC6495. A transient CRISPR strategy was used to generate this strain. The *CaCAS9* cassette was amplified from pLC963 using oLC5974 and oLC5976. The sgRNA fusion cassette was made by PCR amplifying from pLC963 with oLC5978 and oLC6496 (fragment A) and oLC5980 and oLC6497 (fragment B), and then fusion PCR was performed on the fragments using the nested primers oLC5979 and oLC5981. The *RFP-NAT* cassette, sgRNA, and *CAS9* DNA were transformed into CaLC239. NAT-resistant colonies were tested for upstream integration with oLC6526 and oLC3013 and downstream integration with oLC247 and oLC6527. Absence of the wild-type allele was tested with oLC6526 and oLC6527. Expression of the tagged protein was confirmed by microscopy.

The *tetO-DHH1/tetO-DHH1* (CaLC5393) strain was made using a transient CRISPR approach. The promoter replacement cassette was amplified from pLC605 using oLC6778 and oLC6779. The *CaCAS9* cassette was amplified from pLC963 using oLC5974 and oLC5976. The sgRNA fusion cassette was made by PCR amplifying from pLC963 with oLC5978 and oLC6780 (fragment A) and oLC5980 and oLC6781 (fragment B), and then fusion PCR was performed on the fragments using the nested primers oLC5979 and oLC5981. The *NAT-tetO* cassette, sgRNA, and *CAS9* DNA were transformed into CaLC239. Upstream integration was tested using oLC534 and oLC6782, and downstream integration was tested using oLC300 and oLC6783. Lack of a wild-type allele was tested using oLC6782 and oLC6783. The strain was then flipped on YNB-BSA to restore NAT sensitivity. qRT-PCR was performed to check for DOX-mediated transcriptional repression using oLC6905 and oLC6783.

The *tetO-CAM1/tetO-CAM1* strain (CaLC5452) was made using a transient CRISPR approach. The promoter replacement cassette was amplified from pLC605 using oLC6950 and oLC6951. The *CaCAS9* cassette was amplified from pLC963 using oLC5974 and oLC5976. The sgRNA fusion cassette was made by PCR amplifying from pLC963 with oLC5978 and oLC6973 (fragment A) and oLC5980 and oLC6974 (fragment B), and then fusion PCR was performed on the fragments using the nested primers oLC5979 and oLC5981. The NAT-tetO cassette, sgRNA, and Cas9 DNA were transformed into CaLC239 and colonies were selected on YPD + NAT. Upstream integration was tested using oLC534 and oLC6952 and downstream integration was tested using oLC300 and oLC6953. Lack of a wild-type allele was tested using oLC6952 and oLC6953. The strain was then flipped on YNB-BSA to restore NAT sensitivity.

To make the doubly-tagged Ulp1-GFP tagged strains (CaLC5932 and CaLC6033), the Ulp1-GFP-NAT cassette with homology to the Ulp1 locus was amplified from pLC1046 using oLC7542 and oLC7543. The transient CRISPR strategy was used to generate this strain. The *CaCAS9* cassette was amplified from pLC963 using oLC5974 and oLC5976. The sgRNA fusion cassette was made by PCR amplifying from pLC963 with oLC5978 and oLC7540 (fragment A) and oLC5980 and oLC7541 (fragment B), and then fusion PCR was performed on the fragments using the nested primers oLC5979 and oLC5981. The *GFP-NAT* cassette, sgRNA, and *CAS9* DNA were transformed into CaLC239 or CaLC3385. Upstream integration was tested using oLC600 and oLC7545 and downstream integration was tested using oLC274 and oLC7544. Absence of a wild-type copy was confirmed with oLC7567 and oLC7568. The strain was then flipped on YNB-BSA to restore NAT sensitivity. Expression of the tagged protein of the right size was confirmed by western blotting and microscopy.

To make the doubly-tagged Mck1-GFP tagged strains (CaLC5933 and CaLC6032), the *MCK1-GFP-NAT* cassette with homology to the *MCK1* locus was amplified from pLC1046 using oLC7550 and oLC7551. A transient CRISPR strategy was used to generate this strain. The *CaCAS9* cassette was amplified from pLC963 using oLC5974 and oLC5976. The sgRNA fusion cassette was made by PCR amplifying from pLC963 with oLC5978 and oLC7548 (fragment A) and oLC5980 and oLC7549 (fragment B), and then fusion PCR was performed on the fragments using the nested primers oLC5979 and oLC5981. The *GFP-NAT* cassette, sgRNA, and *CAS9* DNA were transformed into CaLC239 or CaLC3385. Upstream integration was tested using oLC600 and oLC7573 and downstream integration was tested using oLC274 and oLC7574. Absence of a wild-type copy was confirmed with oLC7574 and oLC7573. The strain was then flipped on YNB-BSA to restore NAT sensitivity. Expression of the tagged protein of the right size was confirmed by western blotting and microscopy.

### Plasmid construction

Plasmid pLC759 (HSP90 E36A complementation) is based on pLC455 but harbors a putative TRAP mutation in Hsp90 (E36A), introduced by site-directed mutagenesis with primers oLC418 and oLC419 and sequence verified by Sanger sequencing.

Plasmid pLC1046 (GFP-flipper) was made by amplifying a *C*. *albicans GFP* construct from pLC389 with oLC4358 and oLC4386. The construct and pLC49 were digested with KpnI, and then the insert was ligated into pLC49 and transformed into DH5 alpha cells. Colonies were tested for integration and orientation using oLC600 and oLC1785 for upstream and oLC2316 and oLC275 for downstream.

Plasmid pLC1047 (RFP-flipper) was made by amplifying the *C*. *albicans RFP* construct from pLC447 with oLC6004 and oLC4386. The construct and pLC49 were digested with KpnI, and then the insert was ligated into pLC49 and transformed into DH5 alpha cells. Colonies were tested for integration and orientation using oLC851 and oLC1785 for upstream and oLC4416 and oLC275 for downstream.

## Supporting information

S1 Fig GFP-tagged Hsp90 physically interacts with native, untagged Hsp90. Immunoprecipitation of (A) C-terminally and (B) N-terminally GFP-tagged Hsp90 proteins with GFP-binding resin co-purified the untagged Hsp90 protein, while Hsp90 did not co-purify with GFP-binding resin in control cells lacking GFP-tagged Hsp90 (TAP-tagged Hsp90 or Hsp90^E36A^). There was no difference in Hsp90 levels between input samples. Arrows indicate expected molecular weight for tagged and untagged Hsp90 proteins. GFP, green fluorescent protein; TAP, tandem affinity purification.
(TIF)

S2 Fig Alterations in Hsp90 protein levels and *C*. *albicans* morphology in response to Hsp90 inhibition and depletion. (A) Western blotting for Hsp90 levels from *lys2*Δ/*lys2*Δ *tetO-HSP90/hsp90*Δ cells that were untreated, treated with 15 μM GdA to inhibit Hsp90 function, or treated with DOX to repress *HSP90* expression. For DOX treatment, cells were treated with 0.05 μg/mL DOX overnight with a subsequent treatment at 5 μg/mL DOX before harvesting. (B) Morphology of *lys2*Δ/*lys2*Δ *tetO-HSP90/hsp90*Δ cells that were untreated, treated with 15 μM GdA to inhibit Hsp90 function, or treated with DOX to repress *HSP90* expression. For DOX treatment, cells were treated with 0.05 μg/mL DOX overnight with a subsequent treatment at 5 μg/mL DOX before harvesting. DOX, doxycycline; GdA, geldanamycin.
(TIF)

S3 Fig Correlation analysis of Hsp90^E36A^-GFP AP-MS under drug stress. AP-MS of GFP-tagged Hsp90^E36A^was performed on cells grown at 30°C in the presence or absence of (A) 8 μg/mL fluconazole or (B) 100 nM caspofungin. Each dot represents a protein that passed a BFDR cutoff of 0.05. Solid line represents the linear regression, and dotted lines represent the 95% confidence interval. Raw data for this figure can be found in S4 Table. AP-MS, affinity purification mass spectrometry; BFDR, Bayesian false discovery rate; GFP, green fluorescent protein; YPD, yeast extract peptone dextrose.
(TIF)

S4 Fig P-body and stress granule proteins in response to drugs. (A) MIC assays were performed in YPD medium at 30°C for 24 hours, and optical densities at 600 nm were averaged for two biological replicates with two technical replicates each. Percent growth is normalized to the no drug condition. To repress target gene expression, the strains were incubated in the indicated concentrations of DOX. Raw data for this figure can be found in S1 Data. (B) Pab1, Cam1, Dhh1, and Kre30 protein levels do not decrease in response to treatment with antifungal drugs. Western blotting was performed on cells grown at 30°C in the presence or absence of 8 μg/mL fluconazole (fluc) or 100 nM caspofungin (caspo). Cells were grown overnight in YPD and then subcultured with drugs before protein extraction and western blotting. (C) *PAB1*, *CAM1*, *DHH1*, and *KRE30* transcripts do not decrease in response to treatment with antifungal drugs. Cells were grown overnight in YPD and then subcultured in the presence or absence of 8 μg/mL fluconazole or 100 nM caspofungin before RNA extraction and qRT-PCR. Transcript levels were normalized to *PMA1* and *TEF1*. Significance was determined by one-way ANOVA. ** indicates P value <0.01. Raw data for this figure can be found in S1 Data. Caspo, caspofungin; DOX, doxycycline; Fluc, fluconazole; GFP, green fluorescent protein; HA, hemagglutinin, MIC, minimum inhibitory concentration; P-body, processing body; qRT-PCR, quantitative reverse transcription PCR; YPD, yeast extract peptone dextrose.
(TIF)

S5 Fig Dhh1 forms protein aggregates. Images of Dhh1-RFP and Dhh1-GFP cells at 30°C or 39°C. Scale bar is 10 μm. Arrows indicate aggregates. GFP, green fluorescent protein; RFP, red fluorescent protein.
(TIF)

S1 Table AP-MS under basal conditions. (Tab1) significant interactors. Column A: bait protein. Column B: prey protein. Column C: spectral counts in each replicate. Column D: average spectral counts. Column E: number of replicates performed. Column F: spectral counts identified in each replicate from the control pulldown. Column G: BFDR. Column H: *Candida* genome database bait systematic name. Column I: *Candida* genome database prey systematic name. Column J: *S*. *cerevisiae* interaction information from BioGRID and SGD. (Tab2) All identified interactor peptide counts. Column A: bait protein. Column B: prey protein. Column C: spectral counts in each replicate. Column D: average spectral counts. Column E: number of replicates performed. Column F: spectral counts identified in each replicate from the control pulldown. Column G: BFDR. AP-MS, affinity purification-mass spectrometry; BFDR, Bayesian false discovery rate; BioGRID, biological general repository for interaction datasets; SGD, *Saccharomyces* Genome Database.
(XLSX)

S2 Table SILAC intensity and clusters. (Tab1) Peptide intensity in each fraction. Column A: *Candida* genome database systematic name. Column B: protein common name. Column C: protein uniprot accession ID. Column D: description of function. Column E: lysine used for labeling (L indicates light lysine, M indicates medium lysine, H indicates heavy lysine). Column F: treatment condition (untreated, genetic depletion of *HSP90*, pharmacological inhibition of Hsp90). Column G: co-elution cluster ID. Columns H–DW: intensity in each biochemical fraction. (Tab2) Co-fractionation clustering. Column A: co-elution cluster ID. Column B: protein common name. Column C: protein uniprot accession ID. Column D: *Candida* genome database systematic name. Column E: lysine used for labeling (L indicates light lysine, M indicates medium lysine, H indicates heavy lysine). Column F: treatment condition (untreated, genetic depletion of Hsp90, pharmacological inhibition of Hsp90). (Tab3) GO term analysis of the Hsp90 cluster. Column A: GO term ID. Column B: description of GO term. Column C: frequency of representation over background. Column D: genes assigned to a particular GO term. GO, Gene Ontology; SILAC, stable isotope labeling with amino acids in cell culture.
(XLSX)

S3 Table SILAC fold change. Column A: gene name. Column B: number of fractions with 1.5-fold change (untreated versus pharmacological inhibition of Hsp90). Column C: number of fractions with 1.5-fold change (untreated versus genetic depletion of *HSP90*). Column D: pharmacological inhibition of Hsp90 differences in intensity (fraction ID, intensity at that fraction, fold change). Column E: genetic depletion of *HSP90* differences in intensity (fraction ID, intensity at that fraction, fold change). SILAC, stable isotope labeling with amino acids in cell culture.
(XLSX)

S4 Table AP-MS GFP. (Tab1) Significant interactors. Column A: bait protein and treatment condition. NoDrug indicates the sample was untreated, Caspo indicates the sample was treated with caspofungin, and Flu indicates the sample was treated with fluconazole. Column B: prey protein. Column C: spectral counts in each replicate. Column D: average spectral counts. Column E: number of replicates performed. Column F: spectral counts identified in each replicate from the control pulldown. Column G: BFDR. (Tab2) All identified interactor peptide counts. Column A: bait protein and treatment condition. NoDrug indicates the sample was untreated, Caspo indicates the sample was treated with caspofungin, and Flu indicates the sample was treated with fluconazole. Column B: prey protein. Column C: spectral counts in each replicate. Column D: average spectral counts. Column E: number of replicates performed. Column F: spectral counts identified in each replicate from the control pulldown. Column G: BFDR. AP-MS, affinity purification mass spectrometry; BFDR, Bayesian false discovery rate; GFP, green fluorescent protein.
(XLSX)

S5 Table Strains used in this paper. (Tab1) Strain information. (Tab 2) References for the strains.
(XLSX)

S6 Table Plasmids used in this paper. (Tab1) Plasmid information. (Tab 2) References for the plasmids.
(XLSX)

S7 Table Oligonucleotides used in this paper. (XLSX)

S1 Data Raw data for Fig 4, Fig 6 and S4 Fig. (Tab 1) Raw data for Fig 4, monitoring gene expression changes in response to antifungal drug treatment. (Tab 2) Raw data for Fig 4, monitoring gene expression changes in response to transcriptional repression of *HSP90*. (Tab 3) Raw data for Fig 6. (Tab 4) Raw data for S4C Fig. (Tab 5) Summary of the raw transcriptional expression data. (Tab 6) Raw data for Fig 6C and S4A Fig.
(XLSX)

## Abbreviations

ABC: ammonium bicarbonate
AP-MS: affinity purification-mass spectrometry
ARG: arginine
BFDR: Bayesian false discovery rate
CCT: chaperonin containing TCP-1
BioGRID: biological general repository for interaction datasets
DIC: differential interference contrast
DOX: doxycycline
GdA: geldanamycin
GFP: green fluorescent protein
GO: gene ontology
GRACE: gene replacement and conditional expression
HA: hemagglutinin
HIS: histidine
IEX-HPLC: ion exchange–high performance liquid chromatography
IgG: immunoglobulin G
LC-MS/MS: liquid chromatography–tandem mass spectrometry
LIMS: laboratory information management system
MAPK: mitogen-activated protein kinase
MIC: minimum inhibitory concentration
MMS: methyl methanesulfonate
NAT: nourseothricin
OD_600_: optical density at wavelength 600 nm
P-body: processing body
PKC: Protein Kinase C
PVDF: polyvinylidene difluoride
qRT-PCR: quantitative reverse transcription PCR
R2TP: Rvb1-Rvb2-Tah1-Pih1
RFP: red fluorescent protein
SAINTexpress: Significance Analysis of INTeractome
SGD: *Saccharomyces* Genome Database
SILAC: stable isotope labeling with amino acids in cell culture
snoRNP: small nucleolar ribonucleoprotein
SUMO: small ubiquitin-like modifier
TAP: tandem affinity purification
TEV: tobacco etch virus
TPR: tetratricopeptide repeat
YPD: yeast extract peptone, dextrose
