# Supplementary figures and images for: Global proteomic analyses define an environmentally contingent Hsp90 interactome and reveal chaperone-dependent regulation of stress granule proteins and the R2TP complex in a fungal pathogen
Source: PLoS Biol. 2019 Jul 8;17(7):e3000358. doi: 10.1371/journal.pbio.3000358 (PMC6638986; doi:10.1371/journal.pbio.3000358)

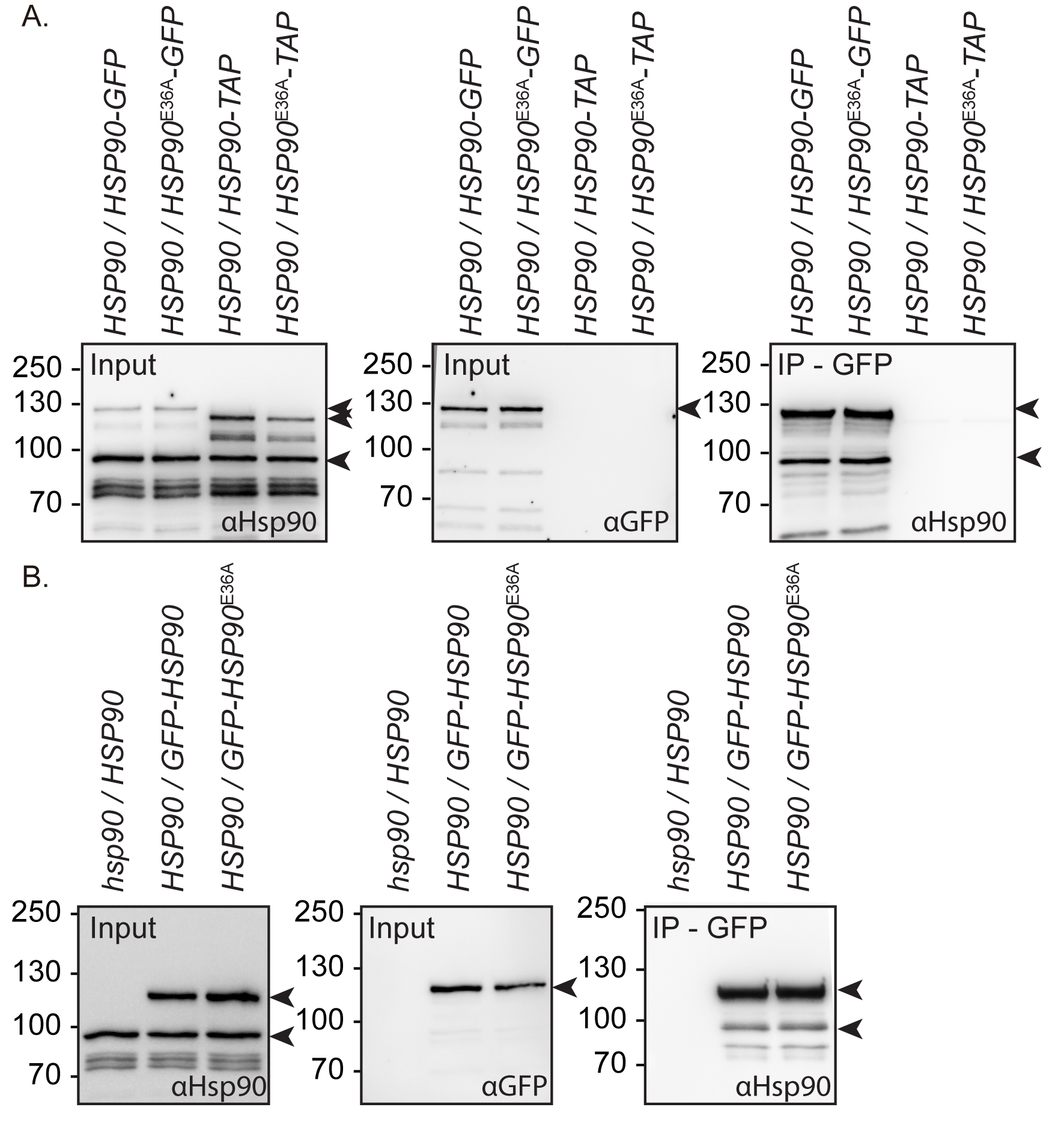

Supplement: S1 Fig — Immunoprecipitation of (A) C-terminally and (B) N-terminally GFP-tagged Hsp90 proteins with GFP-binding resin co-purified the untagged Hsp90 protein, while Hsp90 did not co-purify with GFP-binding resin in control cells lacking GFP-tagged Hsp90 (TAP-tagged Hsp90 or Hsp90E36A). There was no difference in Hsp90 levels between input samples. Arrows indicate expected molecular weight for tagged and untagged Hsp90 proteins. GFP, green fluorescent protein; TAP, tandem affinity purification. (TIF) [file pbio.3000358.s001.tif]

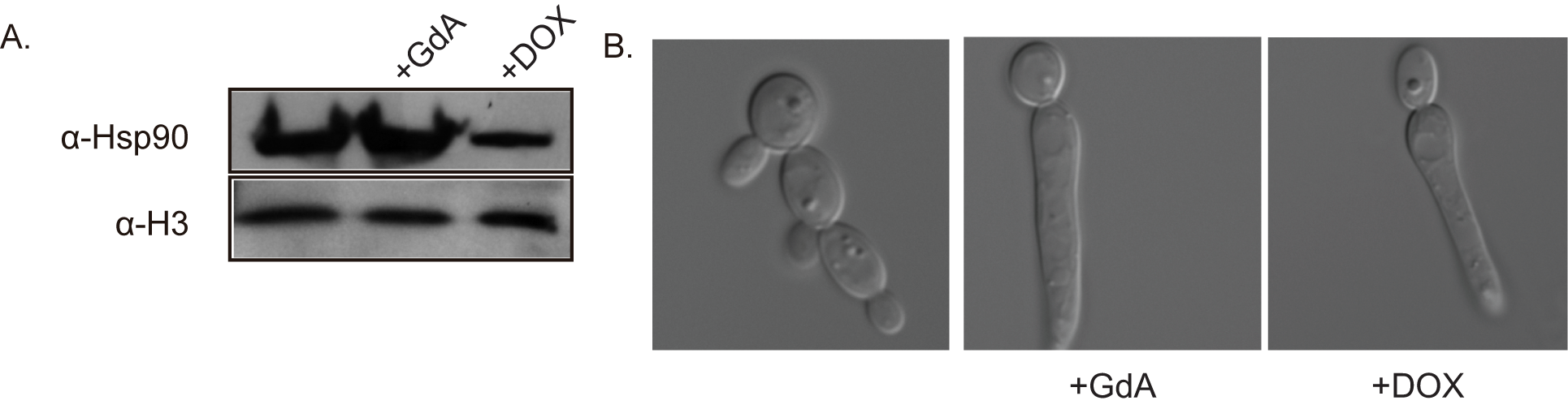

Supplement: S2 Fig — (A) Western blotting for Hsp90 levels from lys2Δ/lys2Δ tetO-HSP90/hsp90Δ cells that were untreated, treated with 15 μM GdA to inhibit Hsp90 function, or treated with DOX to repress HSP90 expression. For DOX treatment, cells were treated with 0.05 μg/mL DOX overnight with a subsequent treatment at 5 μg/mL DOX before harvesting. (B) Morphology of lys2Δ/lys2Δ tetO-HSP90/hsp90Δ cells that were untreated, treated with 15 μM GdA to inhibit Hsp90 function, or treated with DOX to repress HSP90 expression. For DOX treatment, cells were treated with 0.05 μg/mL DOX overnight with a subsequent treatment at 5 μg/mL DOX before harvesting. DOX, doxycycline; GdA, geldanamycin. (TIF) [file pbio.3000358.s002.tif]

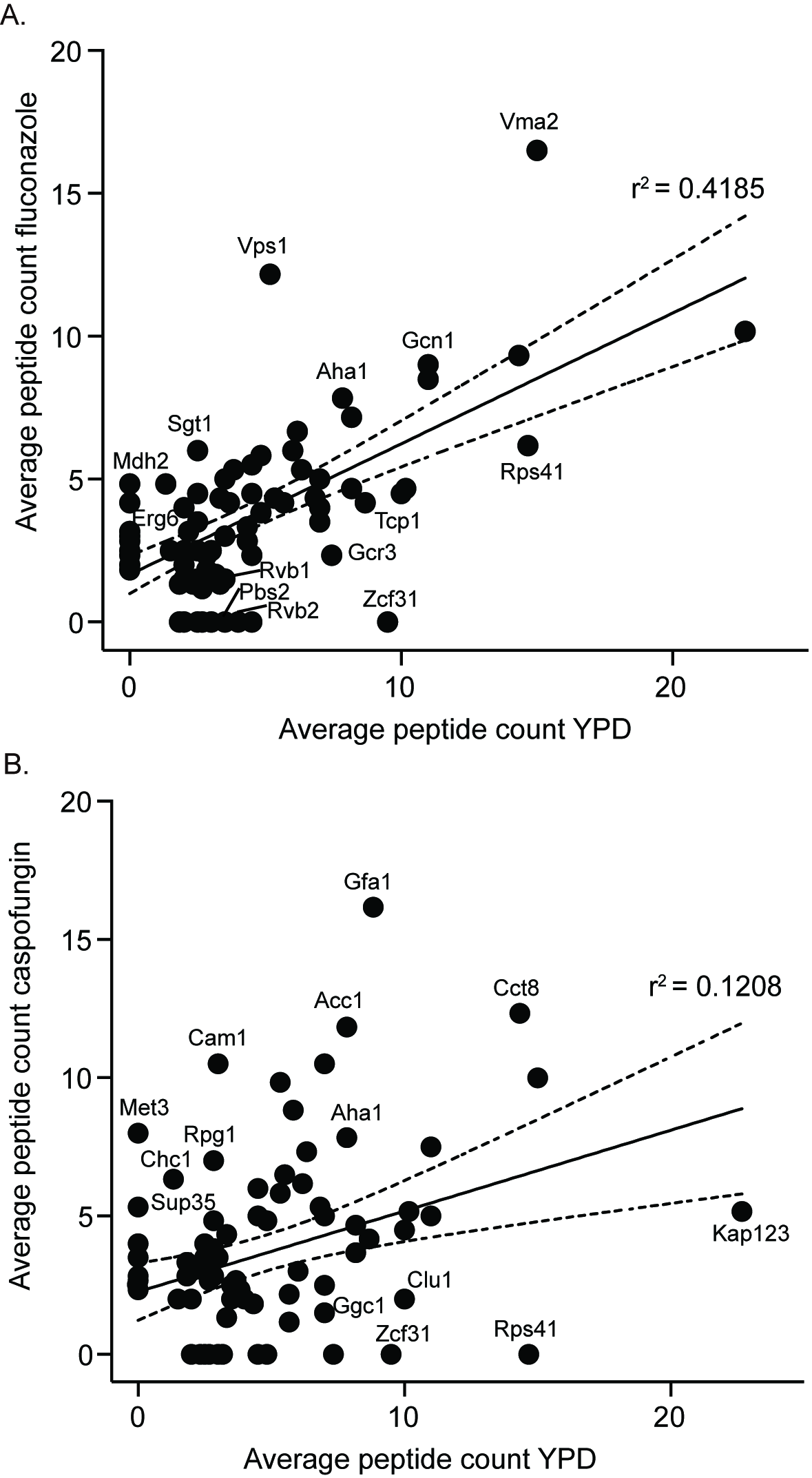

Supplement: S3 Fig — AP-MS of GFP-tagged Hsp90E36Awas performed on cells grown at 30°C in the presence or absence of (A) 8 μg/mL fluconazole or (B) 100 nM caspofungin. Each dot represents a protein that passed a BFDR cutoff of 0.05. Solid line represents the linear regression, and dotted lines represent the 95% confidence interval. Raw data for this figure can be found in S4 Table. AP-MS, affinity purification mass spectrometry; BFDR, Bayesian false discovery rate; GFP, green fluorescent protein; YPD, yeast extract peptone dextrose. (TIF) [file pbio.3000358.s003.tif]

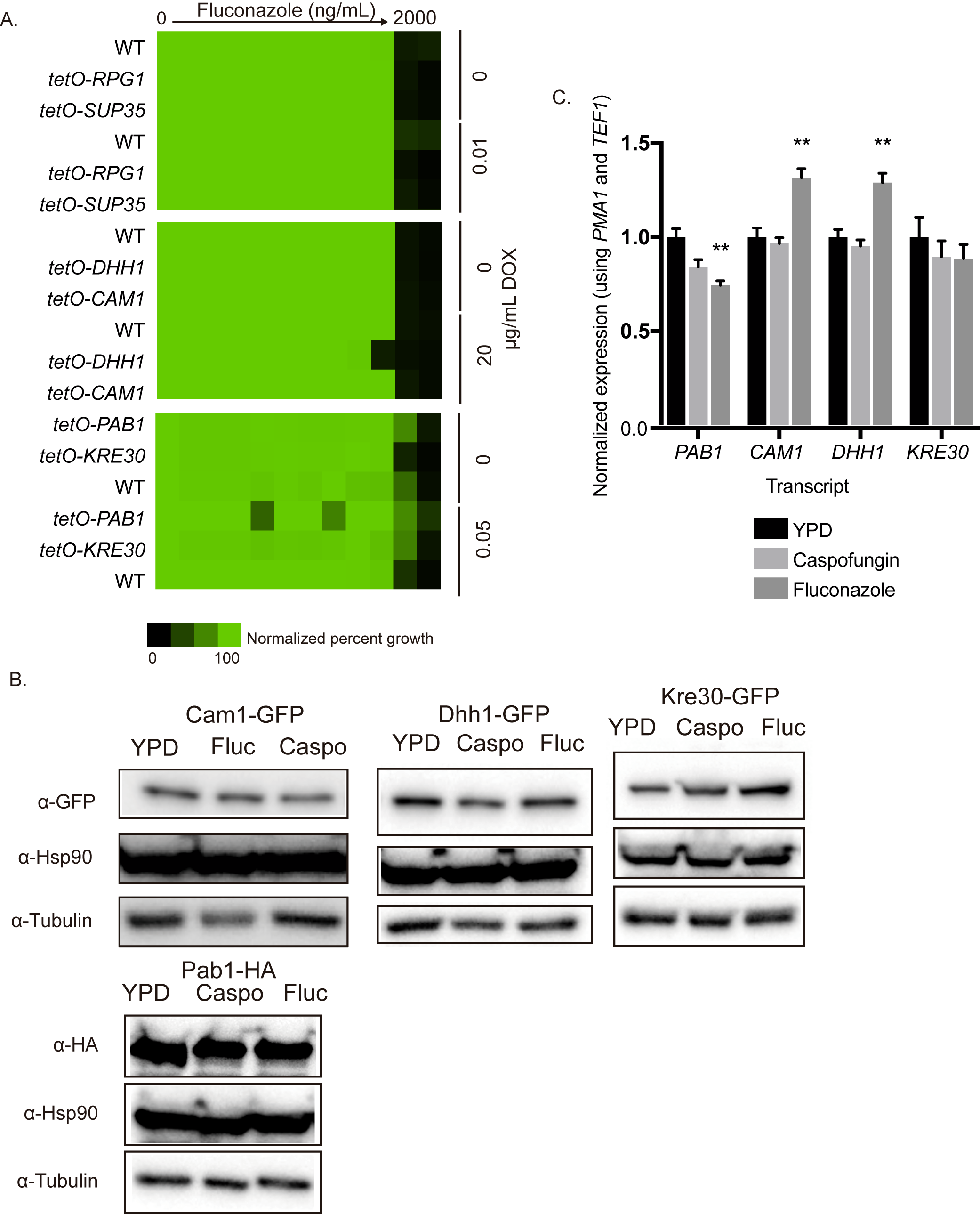

Supplement: S4 Fig — (A) MIC assays were performed in YPD medium at 30°C for 24 hours, and optical densities at 600 nm were averaged for two biological replicates with two technical replicates each. Percent growth is normalized to the no drug condition. To repress target gene expression, the strains were incubated in the indicated concentrations of DOX. Raw data for this figure can be found in S1 Data. (B) Pab1, Cam1, Dhh1, and Kre30 protein levels do not decrease in response to treatment with antifungal drugs. Western blotting was performed on cells grown at 30°C in the presence or absence of 8 μg/mL fluconazole (fluc) or 100 nM caspofungin (caspo). Cells were grown overnight in YPD and then subcultured with drugs before protein extraction and western blotting. (C) PAB1, CAM1, DHH1, and KRE30 transcripts do not decrease in response to treatment with antifungal drugs. Cells were grown overnight in YPD and then subcultured in the presence or absence of 8 μg/mL fluconazole or 100 nM caspofungin before RNA extraction and qRT-PCR. Transcript levels were normalized to PMA1 and TEF1. Significance was determined by one-way ANOVA. ** indicates P value <0.01. Raw data for this figure can be found in S1 Data. Caspo, caspofungin; DOX, doxycycline; Fluc, fluconazole; GFP, green fluorescent protein; HA, hemagglutinin, MIC, minimum inhibitory concentration; P-body, processing body; qRT-PCR, quantitative reverse transcription PCR; YPD, yeast extract peptone dextrose. (TIF) [file pbio.3000358.s004.tif]

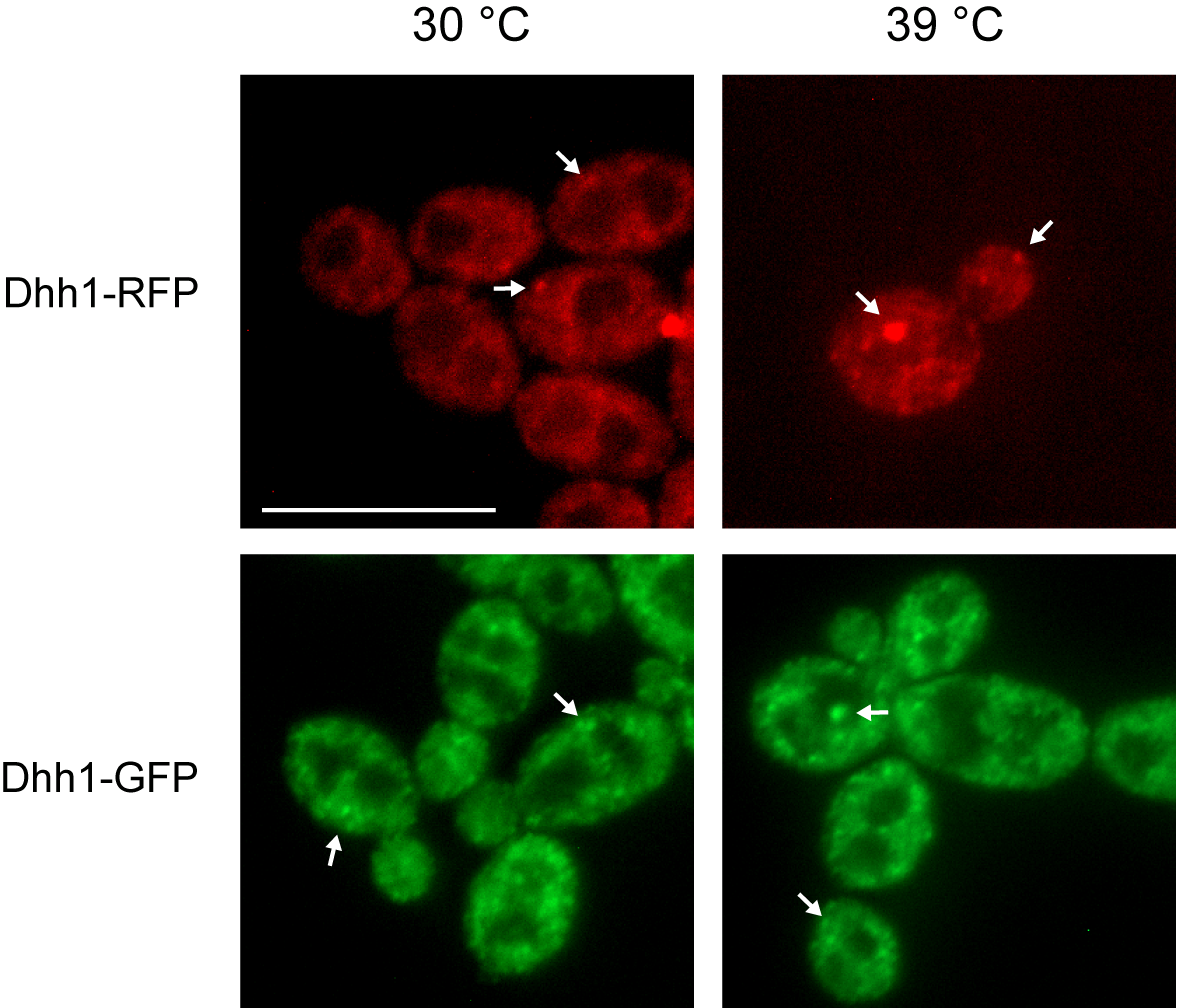

Supplement: S5 Fig — Images of Dhh1-RFP and Dhh1-GFP cells at 30°C or 39°C. Scale bar is 10 μm. Arrows indicate aggregates. GFP, green fluorescent protein; RFP, red fluorescent protein. (TIF) [file pbio.3000358.s005.tif]
